# Hydro-chemical profiling and contaminant source identification in agricultural canals using data driven clustering approaches

**DOI:** 10.1038/s41598-025-08620-z

**Published:** 2025-07-10

**Authors:** Yashaswi Songara, Anupam Singhal, Rahul Dev Garg, Srinivas Rallapalli

**Affiliations:** 1 Department of Civil Engineering, Birla Institute of Technology and Science, Pilani, Rajasthan 333031 India; 2 Civil Engineering Department, Indian Institute of Technology, Roorkee, Dehradun, India

**Keywords:** ANOVA, Canal systems, Cluster analysis, Modeling, Water quality, Environmental sciences, Hydrology, Engineering, Civil engineering

## Abstract

Canal networks are vital for irrigated agriculture in semi-arid regions, yet their water quality is increasingly endangered by diffuse agro-chemical runoff and unregulated effluent discharges. Despite this growing risk, long-term, high-resolution assessments that simultaneously capture spatial patterns and seasonal dynamics remain scarce—leaving practitioners with limited evidence for targeted interventions. Addressing this gap, the study sampled ten canal sites monthly for 11 months across Charkhi Dadri District (Haryana, India) and analysed sixteen physicochemical parameters, including heavy metals and irrigation-relevant ions. A suite of multivariate techniques—R- and Q-mode hierarchical clustering, principal-component analysis (PCA), correlation matrices and one-way ANOVA—was employed to disentangle pollution drivers, while the Irrigation Water Quality Index (IWQI) translated complex chemistry into management-ready scores. Two principal components explained 72.6% of variance, with aluminium, iron and copper emerging as dominant contributors; ANOVA revealed significant seasonal shifts (*p* < 0.05) in these metals. Cluster analysis pinpointed contamination hotspots, and IWQI values of 67.3–85.5 classified canal water as “good” to “very good” for irrigation. By integrating granular spatiotemporal monitoring with advanced multivariate statistics, the study delivers a scalable framework for managing irrigation canals in data-limited, semi-arid landscapes.

## Introduction

Surface water is crucial for maintaining ecological health^1^. The quality of water determines crop growth; it is one of the basic needs for agricultural practices^2^. Before the availability of canals, agriculture activities were dependent on the rainfall. But with increase in population, demand of the food has increased which led to expansion of irrigated areas throughout the world, particularly in arid and semi-arid regions^3^. The increase in agricultural lands in less fertile areas led to advancement in the construction of reservoirs and canal systems^4–6^. Man-made canals are a disturbance to the natural environment. However, with time canals would be valued as natural streams and rivers^7^. Canal system plays a crucial role in irrigation and stormwater management^8^. But the availability of the canal systems increases the accessibility for disposal of wastewater, making surface water bodies more vulnerable to pollution. The wastewater is released into streams due to non-availability of proper sewage drainage channels and the same contaminated water is used for irrigation and other purposes^9^.

Pollutants can enter in surface water due to erosion, heavy runoff, and anthropogenic exploitation which includes mining and agriculture activities which can adversely affect the water quality^10–16^. Any imbalance in quality of water can significantly impact water ecosystems^17,18^.Extensive research has revealed that the properties of surface water, which are vital for irrigation purposes, are significantly influenced by the concentration of major ions^19^. It has also been widely reported that the high heavy metal concentration in the water bodies causes water toxicity^20–23^.

Thus, continuous monitoring of water quality requires analyzing a wide range of physical, chemical, and biological factors, creating a complex framework. Identifying patterns within these attributes and drawing actionable insights is a complex and intricate task^24,25^. There are conventional approaches to assess water quality which involves comparing experimentally determined parameter values. However, these methods often fail to deliver a rapid, holistic view of the water quality. Water quality parameters can be analyzed using other techniques such as modeling, statistical analysis^26,27^. There are various number of parameters because of which difficulty arises to evaluate surface water quality. Hence, Water quality indexes serve as valuable tools for evaluating the overall quality of water. Water quality evaluation relies on a water quality index (WQI), a singular numerical measure obtained through a sophisticated mathematical process that considers parameters related to water quality^28,29^. However, WQI models are biased by some degree of uncertainty produced during the indicator selection process, indicator weighting process^30,31^. Cluster analysis (CA) and Principal Component Analysis (PCA) has been used by many researchers to identify the potential sources of contamination in water bodies^26,32–35^. A long term water monitoring quality program results in a huge amount of data and the uniqueness of PCA and CA is that it can significantly reduce the dataset dimensionality^36,37^. To assess the water quality, hierarchical cluster analysis (HCA), and principal component analysis are suitable for such type of studies. It is used to reveal useful information from the dataset^38^. It assists in understanding and interpreting the association between various variables^39^. PCA is more practical and cost-effective as it reduces the time, effort, and expenses. Beyond its economic advantages, PCA techniques play an important role in environmental management by delivering insights that enable stakeholders to make informed decisions for the sustainable management of surface water systems^40^. Moreover, to group the water quality parameters based on similarities and dissimilarities between various classes ward’s linkage method is an efficient tool^41^. The combined use of the Irrigation Water Quality Index (IWQI) and multivariate statistical methods facilitates efficient water resource management and the rapid identification of solutions to water pollution issues^33^. Analysis of variance (ANOVA) is an efficient method for analyzing experimental data. Also, to find out variation of various parameters ANOVA is used. It is a complex technique with numerous variations, each applies based on specific experimental data^42^. It was originally developed to assess difference among multiple groups and thus avoid the problem of making multiple comparisons between group means^43^. In the context of this study, it investigates the significant spatial and seasonal difference at a probability level of 0.05^44^. Till now, very limited research has been conducted for the seasonal variation which includes 11 months of data of various physicochemical parameters for canal water in the rural region.

The advanced multivariate techniques used in this study provide novel insights into the relationships of the water quality parameters through creation of biplots and dendrograms.The study’s objectives include: 1)assessing sixteen selected physicochemical parameters of the canal water, providing insights into its composition and characteristics; 2) comparing seasonal variations in the water quality of the canal; 3) creating clusters of parameters and associated stations that receive pollutants from similar sources; 4) identifying key contaminants, their sources, and the relationships between them; and 5) conducting a farmer survey focusing on their perceptions of canal water quality across different seasons, including aspects such as smell and appearance, as well as their observations of changes in water quality over the years and their understanding of the impact of poor water quality on crops. The findings will help policymakers by providing a detailed assessment of the canal’s water quality through key physicochemical parameters, enabling informed decisions on water treatment and management. Comparing seasonal variations and identifying pollution sources highlighted critical areas for intervention and pollution control strategies. Additionally, the farmer survey offered insights into local perceptions and awareness, which can guide educational campaigns and policy initiatives aimed at improving water quality and sustainable agricultural practices.

## Materials and methods

While multivariate statistical techniques like PCA and HCA have been applied in previous water quality studies, the present work brings novelty through its integrated, long-term assessment of canal water quality over an 11-month period in a semi-arid agricultural region. The study provides a rare seasonal analysis of 16 physicochemical and heavy metal parameters, capturing dynamic shifts due to rainfall and agricultural runoff—an area with limited prior datasets of such temporal resolution. Technically, the study combines R-mode and Q-mode hierarchical clustering with biplots and scree plots to unravel complex pollutant interrelationships and source linkages, which enhances the interpretability of multivariate outputs in a spatial context. Additionally, the application of irrigation-specific indices (IWQI, SAR, Na%, MH, KI) with component-based statistical weighting offers a more objective and regionally relevant classification of irrigation suitability. The study provides actionable insights for water resource managers in canal-dependent rural systems, enabling targeted pollution control and informed irrigation planning in the face of increasing water quality challenges linked to agricultural intensification and climate variability.

### Study area

The study area, Charkhi Dadri District, is located in Haryana state. It covers an area of 38,706.02 Ha. It experiences a semi-arid climate, with temperatures ranging from 2 °C in winter to 45 °C in summer. The region receives scanty rainfall, with an annual average of 483 mm, primarily occurring during July and August. Geologically, the area is rich in building stones, gypsum, and flexible stones, particularly in Kaliyana Village. Agriculture is a key occupation, with Bajra and Cotton being the dominant Kharif crops, while Wheat and Sarson (mustard) are grown in the Rabi season. The vegetation in the region consists mainly of thorny trees such as Babool, Jandi, and Kair, alongside other species like Neem, Sheesham, and Peepal. The major soil type is sand and sandy loam. Also, Grazing land is limited, and the region has a low forest cover with a land use of 73.41 Ha of forest area and the gross cropped area is 62,654 Ha. The combination of climatic conditions, soil characteristics, and agricultural practices makes this region significant for studying water quality and environmental sustainability. Charkhi Dadri, is a region of significance in terms of agriculture and water resource management. The canal system harnesses water sourced from the Yamuna River, with the majority of it allocated for irrigation purposes. However, the region faces an annual water scarcity problem during summer season, which challenges the sustainability of agricultural activities. The limited availability of water resources makes it essential to manage and optimize water usage for irrigation purposes carefully. This scarcity is particularly prominent during the warmest months of April, May, and June, when the demand for irrigation water significantly increases. During this period, the canal system assumed a pivotal role as the primary source of irrigation, catering to the water requirements of the agricultural lands in the region. The length of the canal is 32.50 km. In the targeted study area, canal water quality is susceptible to multiple potential sources, major ones are agriculture waste, nearby residential areas and small-scale industries. Therefore, conducting regular studies and seasonal variation is crucial to effectively address pollution and safeguard the water quality^45,46^.

The canal water body is influenced by seasonal variations, driven by factors such as agricultural runoff. These variations can significantly impact the physicochemical characteristics of the water, affecting its quality and suitability for agriculture purposes. The 11-month dataset was selected to capture these fluctuations across different seasons, providing insights into how water quality parameters change over time. This duration allows for an in-depth understanding of temporal trends while ensuring the feasibility of data collection and analysis. Also, it has been found out that some local farmers use this water for drinking purposes. Understanding the seasonal variations in this region is crucial for evaluating potential risks and ensuring water safety. The dataset provides a strong foundation for further research, including long-term trend analysis and potential water management strategies. Furthermore, several studies have successfully conducted water quality assessments using one-year datasets^26,47,48^ demonstrating that such a timeframe is sufficient to capture meaningful seasonal trends. By following a similar approach, this study aligns with established research methodologies while providing valuable insights into the specific conditions of the study area.

### Water sampling

**Fig. 1:**
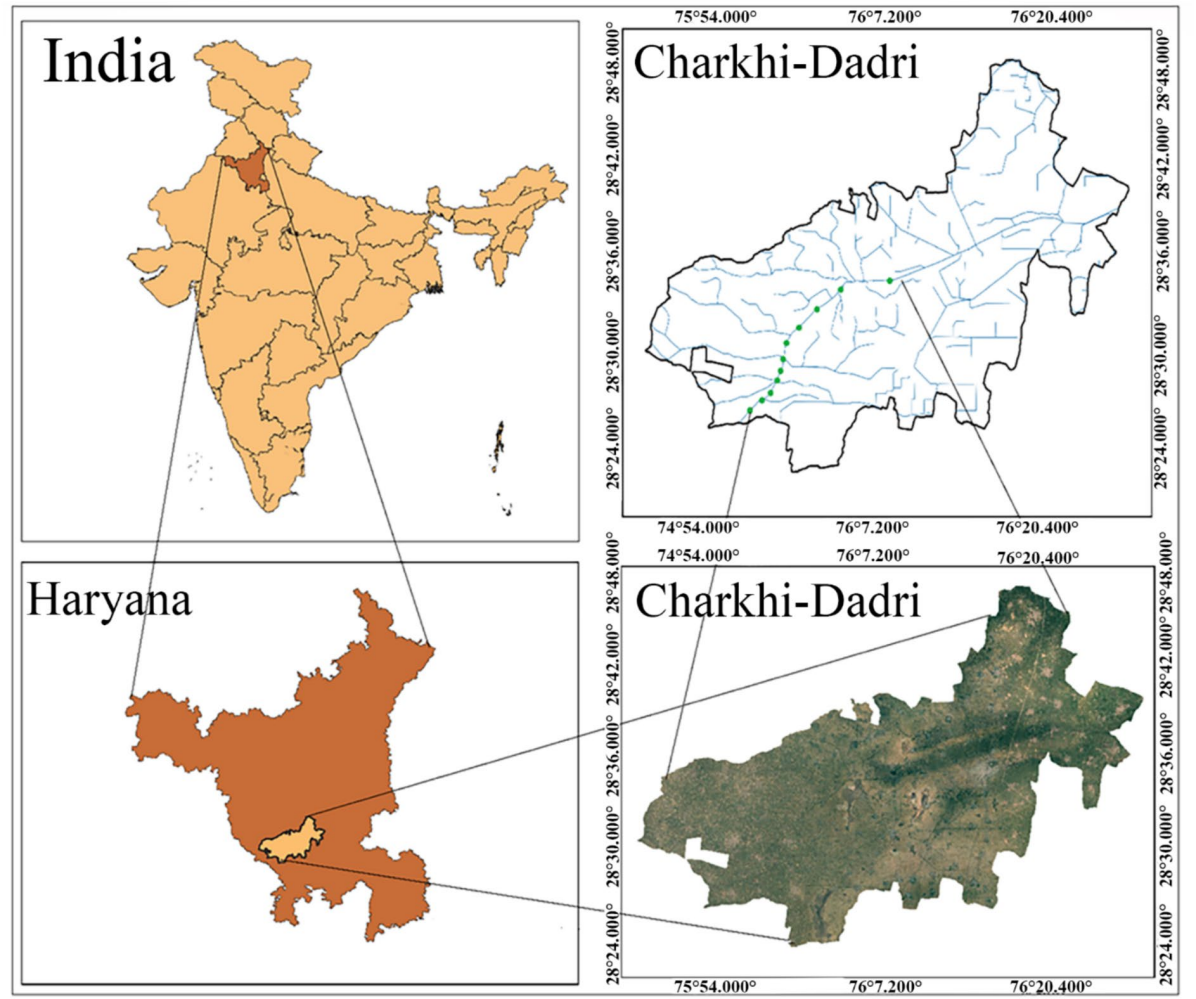
Study area representing the sampling stations (ArcGIS Pro version 3.1, https://pro.arcgis.com/en/pro-app/latest/get-started/download-arcgis-pro.htm ).

**Fig. 2:**
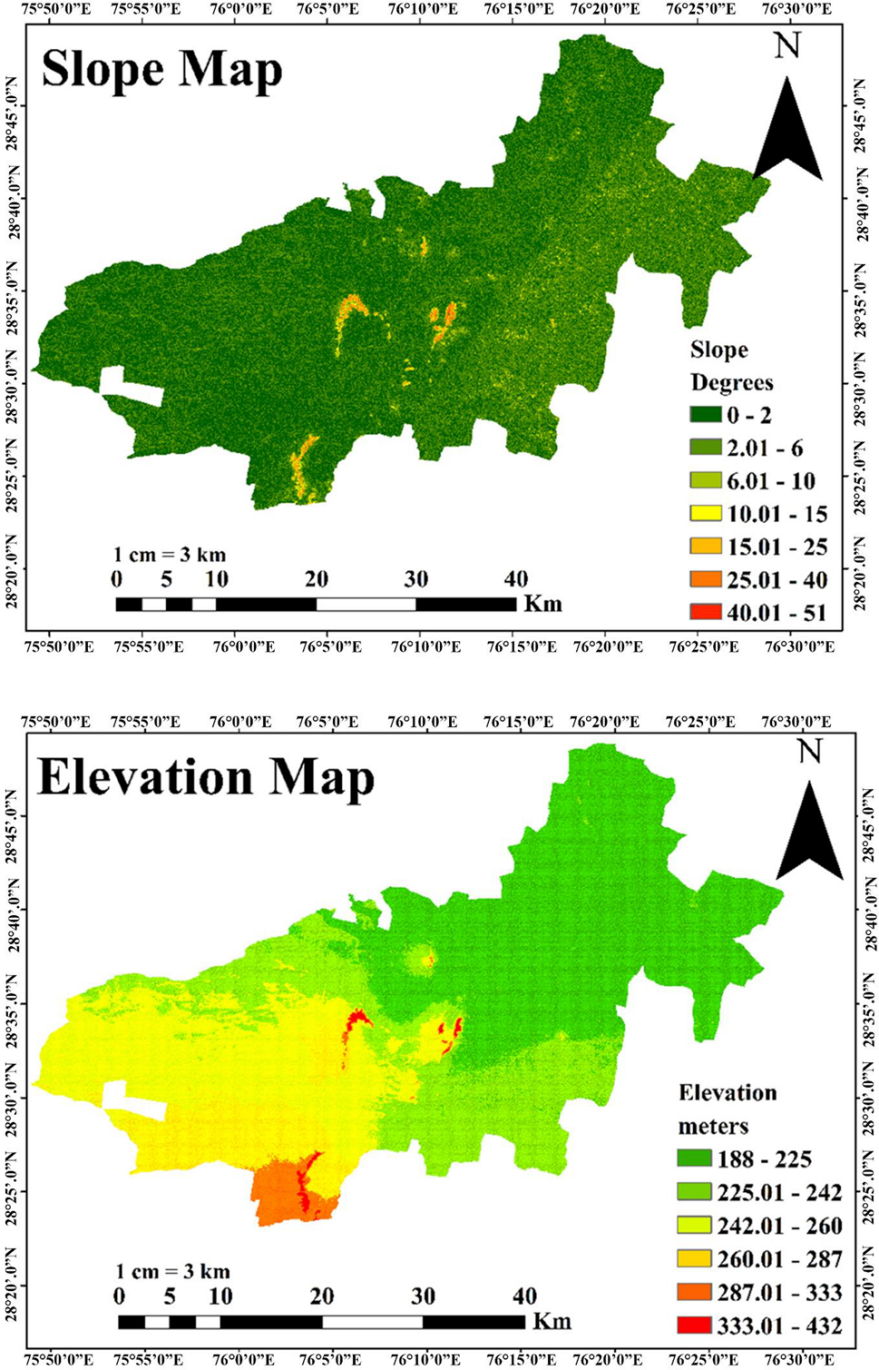
Slope map (top) and DEM (bottom) of the study area (ArcGIS Pro version 3.1, https://pro.arcgis.com/en/pro-app/latest/get-started/download-arcgis-pro.htm).

This study used rigorous water sampling protocols to ensure accurate and reliable data collection. The sampling sites along the Loharu main canal (LMC) were carefully chosen to capture the variability in water quality across different location. Polyethylene bottles were used for sampling. Total ten sites were selected along the Loharu canal system. After the collection, samples were immediately transported to the laboratory within two hours. During the transportation, samples were stored in buckets with ice packs to preserve their freshness and prevent any degradation of water quality parameters. Water samples were collected regularly for 11 months (February 2023 to December 2023) at each sampling site from three points (left corner, right corner and centre). APHA, 23rd editions (2017) were followed for the collection and preservation of samples. The samples were immediately analysed for various physicochemical parameters upon arrival at the laboratory. Throughout the sampling and analysis procedure, the quality control measures were taken to ensure accuracy and reliability. The latitudes and longitudes of the sites are given in Table 1, the locations of the 11 sampling sites are depicted in Fig. 1. Digital elevation model (DEM) and slope map are shown in Fig. 2.

**Table 1:** Precise location of study sites of the canal system.

Sites	Coordinates	
Site 1	28.584°	76.132°
Site 2	28.574°	76.083°
Site 3	28.554°	76.05°
Site 4	28.534°	76.024°
Site 5	28.525°	76.014°
Site 6	28.505°	76.007°
Site 7	28.495°	76.004°
Site 8	28.49°	76.002°
Site 9	28.47°	75.989°
Site 10	28.46°	75.977°
Site 11	28.458°	75.970°

### Water quality characterization

**Fig. 3:**
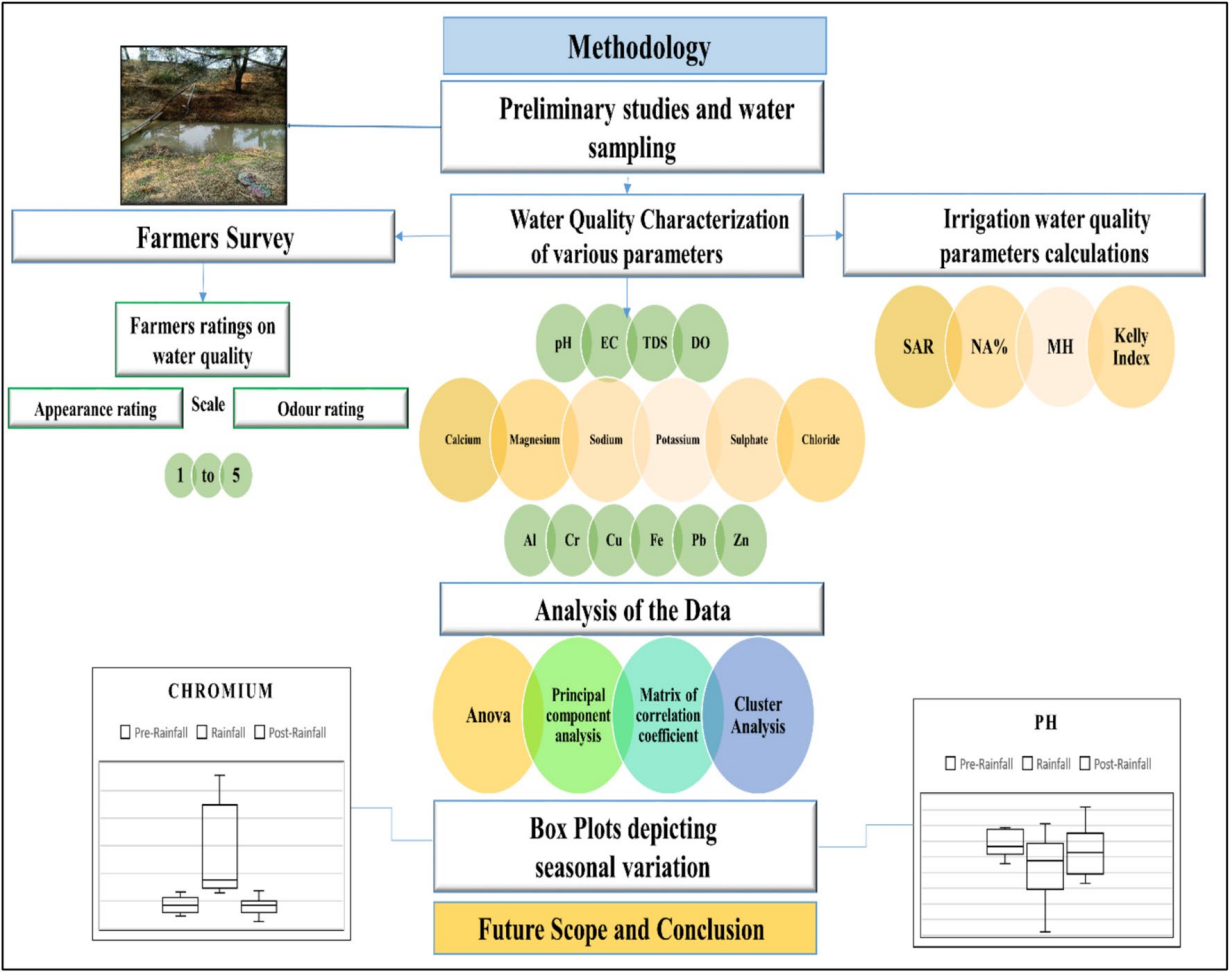
Flowchart representing the schematic procedure adopted in the study.

**Fig. 4:**
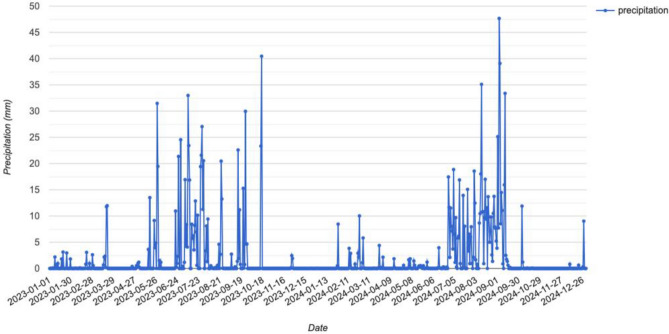
Daily precipitation data for the year 2023–2024.

*Laboratory analysis*: The water samples underwent analysis in the laboratory using American Public Health Association (APHA)23rd editions (2017).Standard methods of analysis of various parameters of water quality were employed to determine chloride (APHA 23rd edn. 2017–4500 ${Cl}^{-}$ B), sulfate (APHA 23rd edn. 2017–4500 -${{So}_{4}}^{2-}$ D) and dissolved oxygen (APHA 23rd edn. 2017–4500-O C). ThepH, electrical conductivity (EC) and total dissolved solids (TDS) was measured using pH meter (OAKION) model PC2700.Furthermore, cations and heavy metals were measured by Inductively Coupled Plasma-Optical Emission Spectrometer, (ICP-OES) Optima 7000DV (with Auto sampler S10 Series), Perkin Elmer. The procedure of the study is given in Fig. 3 with a descriptive flowchart. Figure 4 presents the precipitation data for 2-year period (2023–2024). This comprehensive analysis facilitated a thorough characterization of the water samples, providing valuable insights into their physicochemical composition, thereby enhancing our understanding of water quality and potential environmental impact.

### Data analysis

To find out the seasonal and spatial variation of water quality parameters, one-way analysis of variance (ANOVA) was conducted. Many researchers have used this technique ANOVA for water quality evaluation^49,50^. The two-tailed Spearman rank correlation examined the relationships between water quality parameters. The Spearman correlation coefficient (r), which ranges from −1 to + 1, was used to quantify correlation between parameters. Values range from 0.8 to 1 is considered as a strong relation between the parameters and if the values lies between 0.5 and 0.8 it is considered as a moderate^51,52^. PCA was carried out for water quality analysis. This method reduces the dimensionality of a correlated dataset with minimum loss of information^53^. PCA extracts eigenvalues of correlation matrix, and these eigenvalues indicates the significance of principal components. To find out the principal component loadings (PCs), The eigenvectors calculated in this technique are then multiplied by the square root of eigenvalues, which represents the significance of each variable to a particular component^54^. Eigenvalues which are equal or greater than 1 are considered significant in the analysis^55^. Box plots were also prepared to show a significant seasonal variation of various parameters. To efficiently classify and segregate water quality parameters, cluster analysis is an efficient tools^56^. This technique organizes the samples into clusters signifying hierarchy through dendrogram. Agglomerative and divisive are the two important methods used for clustering of datasets, and dendrogram is used to present organized samples and their respective relations^57^.

### Survey description

The survey was done in two phases and total sample size of participants were approximately 479. Verbal consent was taken from all individuals who participated in the survey. We asked questions to farmers about the perception of canal water, including problems with the smell and appearance of canal water for different season. We then elicited the respondents’ ratings of the canal water around the farmer’s agriculture area in several dimensions: smell and appearance. We then ask about the knowledge they have related to the impact of poor water quality on crops. We also asked the farmers about the awareness they have related to the preservation techniques to improve water quality. The survey part was administered from September 2023 to January 2024, and participants were physically contacted with the following message: "We are surveying on canal water quality. If you would like to take part please help us fill out this form." The response rate of the farmers was around 55%. The questionnaire was given in the below Table 2. Informed consent was obtained from all participants prior to participation in the study.

**Table 2:** Canal water quality ratings obtained from farmers survey in the study area.

Questionnaire Item	Farmers Ratings	
	Mean	Standard deviation*
Water appearance rating of farmers for pre-rainfall period	3.684	0.639
Water appearance rating of farmers for during-rainfall period	3.114	0.844
Water appearance rating of farmers for post-rainfall period	3.231	0.814
Water smell rating of farmers for pre-rainfall period	3.753	0.689
Water smell rating of farmers for during rainfall period	3.526	0.701
Water smell rating of farmers for post-rainfall period	3.716	0.857

Ratings for smell and appearance of canal water on the scale: ‘bad’ (= 1); ‘poor’ (= 2); ‘adequate’ (= 3); ‘good’ (= 4), and ‘excellent’ = (5),

### Irrigation water quality criteria

The assessment of water quality for irrigation involves examining the presence and relative proportions of different elements, which can provide valuable insights into its suitability for agricultural purposes. Several commonly used calculated irrigation quality criteria are calculated to evaluate the irrigation water quality. These criteria include the sodium absorption ratio (SAR), sodium percentage (Na%), magnesium hazard percentage (MH%), and Kelly’s index (KI). These criteria are used to determine the potential impact of water on soil properties and crop growth. The formulations for these criteria are as follows:

*Sodium absorption ratio (SAR)* Increased sodium content in irrigation water heightens sodium hazards and diminishes its suitability for irrigation^58^. The Sodium Adsorption Ratio (SAR) is a widely used parameter to assess sodium hazards in irrigation water^59^. SAR reflects the sodium hazard and is computed using Eq. (1). The elevated levels of sodium badly affect the soil infiltration rate and to maintain the optimal soil properties, the presence of sufficient levels of (${\text{Ca}}^{++}$) and (${\text{Mg}}^{++})$ ions are needed as the soil particles have the ability to uptake ${Na}^{+}$ ions. Sufficient levels of (${\text{Ca}}^{++}$) and (${\text{Mg}}^{++})$ ions availability in the soil counterbalance the effect of ${Na}^{+}$ ions^60,61^.

1 $$\text{Sodium absorption ratio }=\frac{{Na}^{+}}{\frac{\sqrt{({Ca}^{++}+{Mg}^{++}}) }{2}}$$

*Sodium percentage (Na%)* Evaluating ${\text{Na}}^{+}$ in surface water involves, determining the percentage of soluble ${\text{Na}}^{+}$ ions in the water. The ${\text{Na}}^{+}$ percentage is a widely employed statistical method to assess the suitability of natural waters for irrigation, as it reflects the interaction between ${Na}^{+}$ ions and their influence on soil permeability^62^. Excessive ${Na}^{+}$ accumulation in the water can lead to soil degradation. Soils with elevated levels of ${(\text{Na}}^{+}$ and ${{\text{CO}}_{3}}^{-}$) exhibit alkaline properties, alkaline soils have a pH above seven and are characterized by poor soil structure. The high sodium content interferes with the soil’s ability to retain water and nutrients, leading to decreased plant productivity, whereas soils with high ${Na}^{+}$ and ${Cl}^{-}$ concentration are classified as saline soils. Saline soils have elevated levels of salt content, and excessive salt accumulation affects the osmotic potential of the soil, making it difficult for plants to extract water. This can lead to water stress in plants and inhibit their growth^63,64^. Na% is evaluated using Eq. (2):

2 $$\text{Sodium percentage }=\frac{{(Na}^{+}+{ K}^{+})}{ ({Na}^{+}+{K}^{+}+ {Mg}^{++} + {Ca}^{++})}\times 100$$

*Magnesium hazard (MH)* Elevated levels of magnesium ${(Mg}^{++}$) in irrigation water can harm the health of soil and plants. Excess magnesium content can adversely affect the structure and permeability of the soil. High-level magnesium can cause soil particles to disperse, resulting in poor soil structure and reduced water infiltration^65^. Additionally, it can contribute to soil compaction, reducing the ability of water to pass through^66^.Moreover, high levels of magnesium can disrupt the uptake of essential nutrients such as calcium (Ca), potassium (K), and ammonium (NH4 +), thereby affecting their availability to plants^67^. Imbalances in the ratio of magnesium to calcium can lead to calcium deficiency symptoms in plants^68^. High magnesium levels in irrigation water can significantly affect soil and plant health, impacting soil structure, nutrient availability, and crop productivity. MH is calculated using Eq. (3):^69^

3 $$\text{Magnesium hazard}=\frac{{(Mg}^{++})}{ ({Mg}^{++} + {Ca}^{++})}\times 100$$

### Kelly’s index (KI)

Kelly’s Index is a parameter for assessing irrigation water suitability in agriculture. The formula of the Kelly index is shown in Eq. (4). It evaluates the risks of high sodium concentrations, which can negatively affect soil properties and crop productivity. A value below 1 indicates safe irrigation, while values above 1 indicate a higher risk of soil degradation^70,71^. Kelly’s Index is an essential tool for making informed decisions in water management strategies, considering its impact on soil quality and crop production. Kelly’s Index is calculated using:^62^

4 $${\text{Kelly}}'{\text{s}}\,{\text{index}} = \frac{{({\text{Na}}^{ + } )}}{{ \left( {{\text{Mg}}^{ + + } + {\text{Ca}}^{ + + } } \right)}}$$

### Irrigation water quality index (IWQI)

The IWQI is an important parameter for determining impact on water quality and thus the suitability of the water for irrigation purposes^72,73^. The IWQI was divided into five categories: (i) excellent (85–100), (ii) very good (70–85), (iii) good (55–70), (iv) satisfactory (40–55), and (v) inappropriate (0–40) (Table 3). Computation begins with principal component and factor analysis (PC/FA) was used to identify the parameters that contribute to the variability of water irrigation. These variables are determined by these factors, and the large dataset is reduced for easy interpretation. The Kaiser–Meyer–Olkin index (KMO), which should be greater than 0.5, and the Bartlett sphericity test were used to determine the adequacy of the selected factors^74^. Using Eq. (5), the weight values (w_i_) were normalized and their final sums equalled one^75^.

5 $${W}_{i}=\frac{\sum_{j=1}^{k}{F}_{j}{A}_{ij}}{\sum_{j=1}^{k}\sum_{i=1}^{n}{F}_{j}{A}_{ij}}$$

where w_i_ is the parameter’s weight, F is the component 1 autovalue, A_ij_ is the explainability of parameter I by factor j, i is the number of physical–chemical parameters chosen by the model, ranging from 1 to n, and j is the number of factors chosen in the model, ranging from 1 to k. The Irrigation water quality was then calculated using Eq. (6)

6 $${{q}_{i}=q}_{max}-\frac{{(X}_{ij}{-X}_{\text{inf}})\times {q}_{iamp}}{{X}_{iamp}}$$

where, q_max_ is the maximum value of q_i_ for the class, X_ij_ is the observed value, X_inf_ is the lower limit of the class to which the parameter belongs, q_iamp_ is the class amplitude, and X_iamp_ is the class amplitude to which the parameter belongs. The X_amp_ of the final class of each parameter was calculated by taking the highest value of physicochemical sample analyses as the upper limit. Equation (7) was used to calculate the IWQI.

**Table 3:** The range and type of irrigation water for IWQI.

IWQI Range	Water type
85–100	Excellent
70–85	Very good
55–70	Good
40–55	Satisfactory
0–40	Unsuitable

7 $$\text{IWQI }= \sum_{i=1}^{n}{Q}_{i}{W}_{i}$$

## Results and discussions

### Experimental analysis of water quality parameters

*Total dissolved solid (TDS) and electrical conductivity* Total dissolved solid (TDS) concentration for the post-rainfall period was 250.74 mg/L and for the pre-rainfall period was 284.10 mg/L. Tables 4 and 5 shows the mean, maximum and minimum values of TDS. With test of significant (*p* < 0.05), the analysis of variance (ANOVA) indicated a significant seasonal variation (*p* value = 0.00). One more study reported a significant seasonal variation between TDS values with *p* < 0.05^55^. Different trend was observed for the TDS values which are 2304.5 mg/L and 840.3 mg/L for the rainfall and summer season^76^which is much higher than the values observed in this study. The mean values of TDS recorded in a study by Lemessa et al.^77^is ranging from 155.3 to 389.67 mg/L. The highest concentrations of TDS may be a result of dissolved solids in the canal system due to agriculture activities, i.e., generating wastewater from the agriculture field.

**Table 4:** Descriptive statistics of physiochemical parameters of study sites.

Sites		pH	EC µS ${cm}^{-1}$	DO mg ${L}^{-1}$	TDS mg ${L}^{-1}$	
Site 1	Mean	7.44	363.88	5.83	273.59	
Site 2	Mean	7.28	361.43	6.04	258.68	
Site 3	Mean	7.21	371.70	5.94	253.60	
Site 4	Mean	7.42	353.39	6.04	255.30	
Site 5	Mean	7.43	365.94	6.09	259.22	
Site 6	Mean	7.44	346.69	6.06	250.89	
Site 7	Mean	7.37	350.00	5.95	260.88	
Site 8	Mean	7.29	365.77	5.96	258.87	
Site 9	Mean	7.34	351.09	5.92	256.27	
Site 10	Mean	7.37	348.57	5.88	246.26	
Site 11	Mean	7.56	387.56	6.12	289.45

**Table 5:** Summary statistics of physical, chemical and qualitative parameters.

Parameters		Mean	Max	Min	S.D*
${Ca}^{++}$	mg ${L}^{-1}$	33.12	36.6	21.34	4.32
${Mg}^{++}$	mg ${L}^{-1}$	14.44	16.53	11.634	1.37
${Na}^{++}$	mg ${L}^{-1}$	16.92	18.92	15.19	1.14
${K}^{++}$	mg ${L}^{-1}$	2.62	3.16	1.637	0.47
${Cl}^{-}$	mg ${L}^{-1}$	35.94	40.67	28.89	3.16
${{So}_{4}}^{-}$	mg ${L}^{-1}$	37.06	40.50	30.84	3.24
pH	mg ${L}^{-1}$	7.35	7.44	7.21	0.08
EC	µS/${cm}^{-1}$	360.55	387.56	346.69	11.72
DO	mg ${L}^{-1}$	5.98	6.12	5.83	0.09
TDS	mg ${L}^{-1}$	260.27	289.45	246.26	11.30
${Al}^{++}$	mg ${L}^{-1}$	1.272	1.501	0.715	0.207
${Cr}^{++}$	mg ${L}^{-1}$	0.025	0.042	0.014	0.009
${Cu}^{++}$	mg ${L}^{-1}$	0.079	0.160	0.017	0.034
${Fe}^{++}$	mg ${L}^{-1}$	1.193	1.428	0.773	0.177
${Pb}^{+}$	mg ${L}^{-1}$	0.048	0.104	0.024	0.021
${Zn}^{++}$	mg ${L}^{-1}$	0.350	0.490	0.168	0.088
SAR	–	3.482	3.895	3.136	0.225
%Na	–	29.245	34.018	26.818	2.013
MH	–	30.521	35.274	26.814	2.420
KI	–	0.359	0.466	0.313	0.042

It has been found out that electrical conductivity EC values for irrigation purposes, 2250 µS/cm is considered suitable, but for highly clayey soil condition, it is not suitable^78^. According to Richards^61^, 750 µS/cm is the ideal value that can be considered. In this study, the EC measurements obtained from the monitored sites conform to the irrigation water EC standards established by the Food and Agriculture Organization (FAO). The mean value of EC for the pre-rainfall period was 377.69 μS/cm and the post-rainfall was 348.77 μS/cm. In one study, opposite trends were observed where the mean values of the study for the pre–rainfall period was 784 μS/cm and post-rainfall period was295.2 μS/cm^79^. While similar trend was observed in one finding where for the rainfall and dry season the value of EC252 μS/cm and 235 μS/cm was reported^80^ which is close to the findings of this study (Table 6).

**Table 6:** Descriptive statistics of cation and anion of the study area.

Sites		Cations mg ${L}^{-1}$				Anions mg ${L}^{-1}$	
		${Ca}^{++}$	${Mg}^{++}$	${Na}^{+}$	${K}^{+}$	${Cl}^{-}$	${{So}_{4}}^{-}$
Site 1	Mean	34.15	12.51	16.71	3.10	28.89	30.85
Site 2	Mean	34.56	14.52	18.03	3.16	33.79	32.46
Site 3	Mean	36.60	15.20	17.32	2.99	36.29	35.58
Site 4	Mean	36.41	15.69	17.43	2.94	39.63	34.09
Site 5	Mean	35.63	14.47	15.70	2.66	40.67	40.51
Site 6	Mean	32.42	16.53	17.22	2.67	38.44	39.31
Site 7	Mean	30.83	14.27	15.19	2.26	37.57	39.95
Site 8	Mean	36.34	14.48	17.60	2.85	34.34	39.26
Site 9	Mean	32.92	14.27	18.92	2.56	35.66	39.46
Site 10	Mean	33.22	15.33	16.73	2.03	36.87	39.97
Site 11	Mean	21.34	11.63	15.36	1.63	33.26	36.32

The seasonal variation in TDS and EC highlights the dual influence of natural hydrological cycles and human-induced inputs from agricultural practices. The elevated TDS values during the pre-rainfall period indicate accumulation of salts and dissolved ions due to evapotranspiration and reduced flow, while the decline post-rainfall points to dilution by stormwater inflows. Although current EC levels remain within permissible irrigation limits, any gradual rise over time could pose threats to soil permeability, especially in clay-dominated soils. This finding underscores the need for periodic assessment of salt loading in irrigation return flows and emphasizes the importance of integrating water-saving irrigation techniques like drip or sprinkler systems to reduce leaching risks and salt buildup in root zones.

*pH and Dissolved Oxygen (DO)* The pH level, an essential parameter for assessing water suitability, is considered in the water quality assessment. The mean pH for the pre-rainfall period was 7.27 and the post-rainfall was 7.24. Overall, during the summer period, the pH values of the collected water samples were found within the given limit for irrigation purposes while for the rainy season, it has been observed that there were fluctuations in pH values. The changes in pH values in this season could attributed to the influence of agricultural effluents which reached in the canal water due to heavy rainfall. It also reveals no substantial variation in pH among the different sites. However, Table 7 highlights substantial variations in pH between different seasons. Past reported pH values in few studies for different season lies between 6.5 and 8.65^80,81^. Aydin et al.^82^ reported the pH value ranged from 7.77 to 9.8 which were slightly alkaline. In one more study, the pH values of the canals water range from 7.9 to 10.96 which showed high alkalinity, with an average value of 8.983 which were not close to results of this study. Ahmed et al.^84^ observations indicated that pH values in the water body lies between 7.2 and 8.0 which were close to the values observed in this study.

**Table 7:** ANOVA values representing the seasonal variation of water quality parameters.

Parameters	Df	F	Sig
pH	2	14.391	0.000
EC	2	5.760	0.008
DO	2	2194.30	0.000
TDS	2	22.64	0.000
${\text{Al}}^{++}$	2	170.44	0.000
Fe	2	95.22	0.000
${\text{Pb}}^{+}$	2	0.138	0.870
Cr	2	11.82	0.000
${\text{Cu}}^{++}$	2	8.10	0.001
${\text{Zn}}^{++}$	2	4.76	0.000
${\text{Ca}}^{++}$	2	7.79	0.002128
${\text{Mg}}^{++}$	2	4.82	0.016
${\text{Na}}^{+}$	2	47.87	0.000
${\text{K}}^{+}$	2	4.31	0.023
${\text{Cl}}^{-}$	2	0.363	0.698
${{\text{So}}_{4}}^{-}$	2	6.436	0.005

**Fig. 5:**
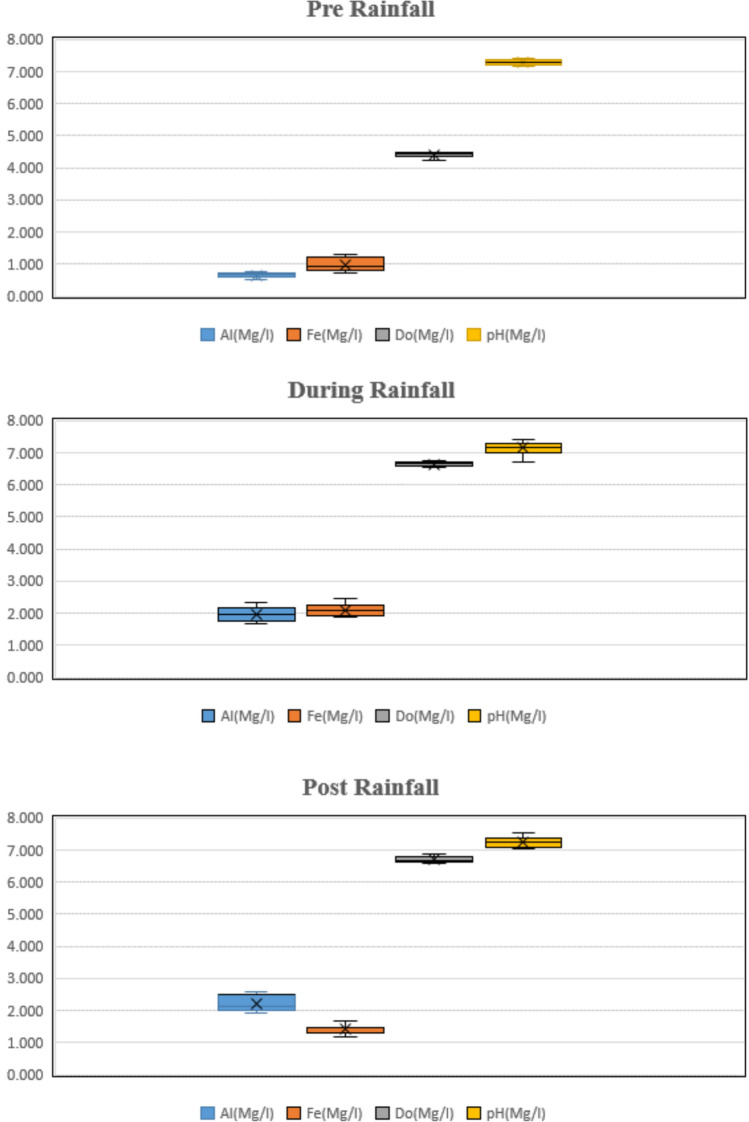

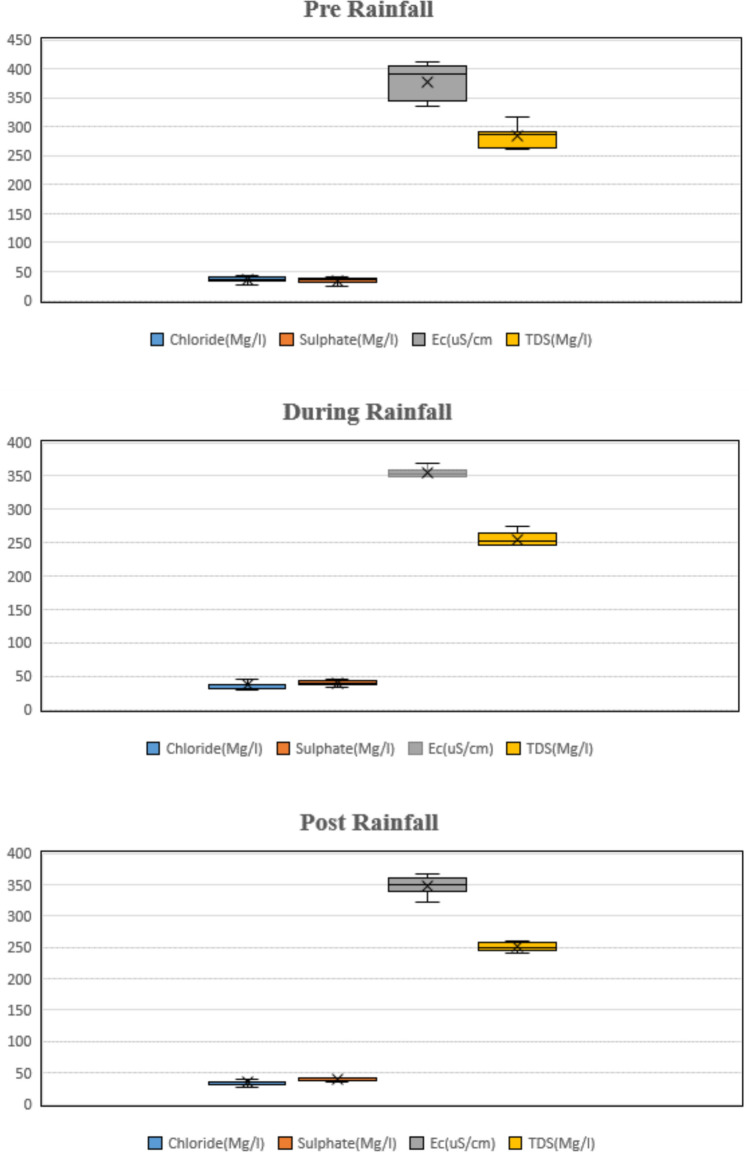

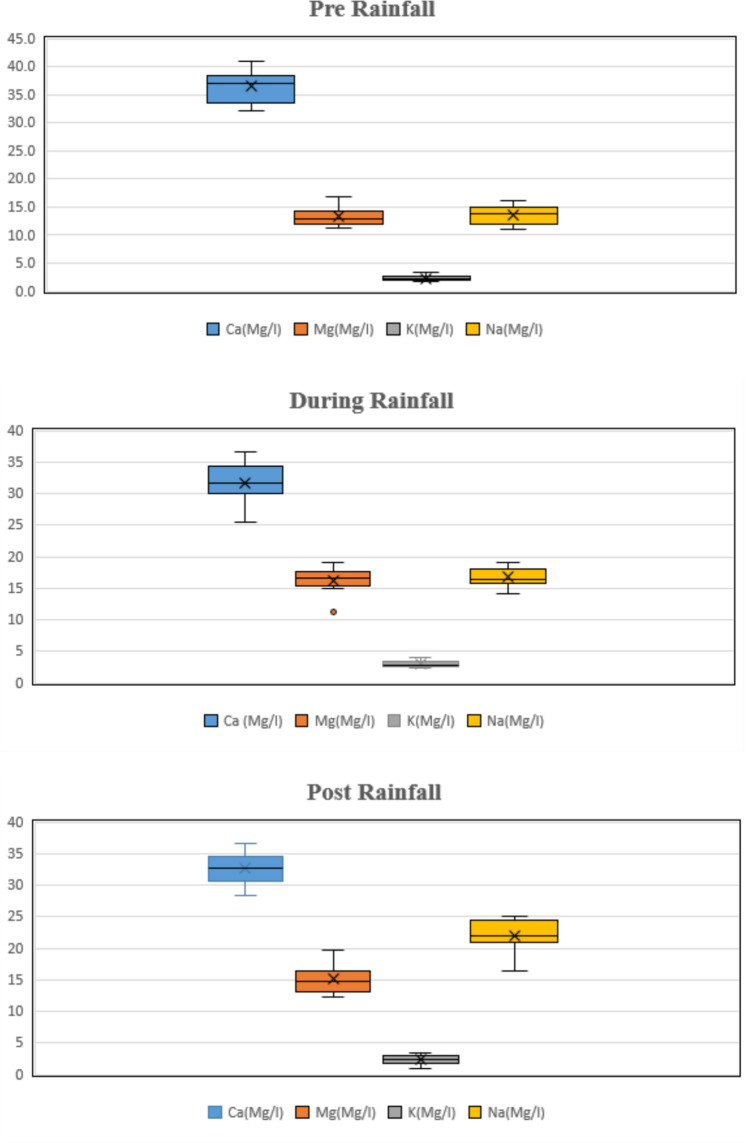

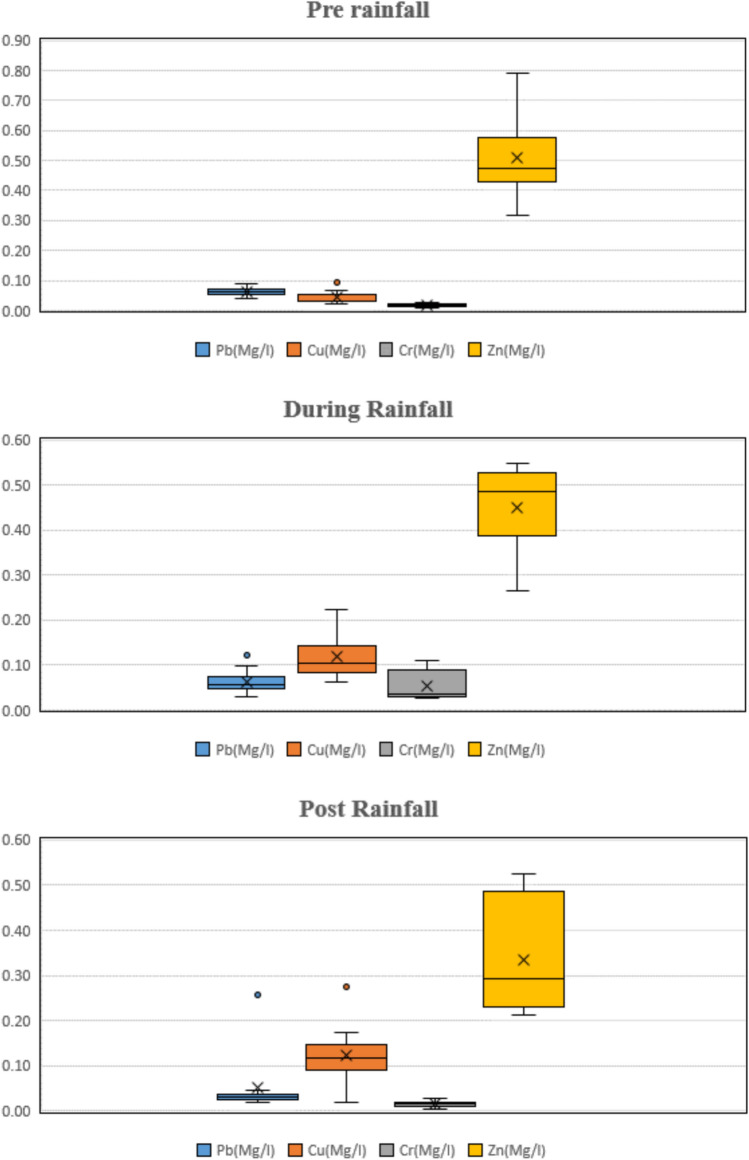
Boxplots of the parameters showing statistically significant seasonal variation.

The spatial distribution and ecological balance of the study area is intricately governed by the concentration of dissolved oxygen (DO). The mean pre-rainfall dissolved oxygen value was 4.40 mg/L and the post-rainfall occurrence was 6.70 mg/L. Remarkably, substantial seasonal variations were detected, underscoring the impact of dynamic environmental conditions as shown in Fig. 5. Specifically, during the summer season, the DO levels experienced a decline probably due to heightened temperatures. Similar observation was found in one study by Ben-Gal et al.^85^ where high temperature might be affecting the DO level in water bodies. Also, other studies follow the similar trend where dissolve oxygen ranging from 1.2 to 6.8 mg/L^81^.

The slight seasonal variation in pH, though within the safe range for irrigation, reflects the buffering response of the canal system to episodic chemical loads, possibly from fertilizers and organic residues. More notably, the significant seasonal shifts in DO suggest strong sensitivity to temperature and organic oxygen demand, with low DO during summer signalling organic pollution stress or reduced flow velocity. These conditions can lead to hypoxic environments that are detrimental to aquatic biota and can reduce microbial-mediated nutrient transformations critical to maintaining water quality. Management strategies should include enhancing canal flow regulation and preventing organic waste discharge, particularly during low-flow seasons, to safeguard aquatic health and maintain oxidation–reduction balance.

The observed changes in pH values during the rainy season can be attributed to agricultural effluents reaching the canal due to surface runoff intensified by heavy rainfall. During rainfall events, fertilizers (such as urea, ammonium nitrate, and superphosphates) and pesticides applied on agricultural fields are washed into the canal system. These substances can alter the chemical composition of the water by introducing acidic or alkaline compounds, depending on the types and concentrations of inputs. For instance, nitrogen-based fertilizers may increase ammonia and nitrate levels, which in turn can lower or raise the pH depending on the buffering capacity of the water. Additionally, organic matter and sediment carried with the runoff can undergo microbial degradation, releasing carbon dioxide and organic acids, further influencing the pH levels.

*Calcium, magnesium, sodium and potassium* The chemical properties of the surface water is primarily influenced by concentrations of major ions^19^. The measured values of calcium (${Ca}^{++}$), magnesium (${Mg}^{++}$), and sodium (${Na}^{+}$) ions in the study area were found to be within the acceptable limits set by the Food and Agriculture Organization. The mean pre rainfall period calcium concentration was 36.47 mg/L and the post rainfall period concentration was 32.63 mg/L given in Table 6. The mean pre rainfall magnesium concentration was 13.31 mg/L and the post rainfall season concentration was 15.15 mg/L. Magnesium and calcium concentration in water bodies vary due to several factors which includes soil type, plant cover, seasonal variation, land type^86^. The values of magnesium observed in a study by Isaac et al.^87^ indicated that there is no significant variation during different season. The pattern observed in the study aligns with the trend observed in this research. The mean pre rainfall sodium concentration was 13.63 mg/L and the dry season concentration was 22.05 mg/L. For the cation Na^+^ value, the findings indicated levels ranging from 1483.88 to 6203 mg/L. Na^+^ levels above 900 mg/L in 70% of the water samples tested during our investigation, which was an issue of concern by the FAO^88^. Potassium (${K}^{+}$) concentrations observed in the study area were not within the FAO permissible limit (2 mg/L). In one more study, it has been observed that the potassium values were not within the permissible limit^55^ (Amer and Mohamed,^51^). The mean pre rainfall potassium (${K}^{+}$) concentration was 2.29 mg/L and the post rainfall season concentration was 2.35 mg/L. Also, a strong negative correlation was observed between K^+^ and.

${{So}_{4}}^{2-}.$ The results of two-tailed Spearman rank correlation are provided in Table 12. Alsubih et al.^88^ observed the cation tendency with the highest value of sodium Na^+^  > K^+^  > Ca^2+^  > Mg^2+^. In one more distributary of Yamuna River, a study was conducted for the parameters which includes calcium, magnesium and it highlighted the need for continuous water monitoring in the region^89^.

The ionic composition of canal water, especially the balance between calcium, magnesium, sodium, and potassium, is crucial for evaluating irrigation suitability and soil health. The generally acceptable levels of calcium and magnesium affirm the water’s potential to maintain soil structure, but elevated sodium and potassium concentrations — especially those exceeding FAO thresholds — raise concern about long-term sodicity and nutrient imbalance. Excess sodium can deteriorate soil porosity, while elevated potassium may interfere with the uptake of other essential nutrients. These findings emphasize the need for monitoring soil exchangeable sodium percentage (ESP) and adopting soil amendments (e.g., gypsum) or alternate water sources where necessary to prevent soil degradation in irrigated regions.

*Sulphate and chloride* Chloride concentration for the mean wet season was 35.60 mg/L and for the dry season concentration was 34.50 mg/L. There is no significant difference between different season (Rainfall and Post-rainfall) observed in the analysis of variance (ANOVA) results. The mean value of Chloride concentrations recorded was 56.84 mg/L in one studyAmer and Mohamed^55^ which is comparatively close to the mean values of this study. Islam et al.^90^ also reported values of chloride ranging from 80.74 to 119.7 mg/L which has not aligned with the results of this study. Whitehead et al.^91^ indicated that the potential source of chloride in water body might be due to chlorinated pesticides.

The mean value of sulphate for rainy season was 39.92 mg/L and the dry season concentration was 34.15 mg/L. The analysis of variance (ANOVA) showed no notable difference between different sites. In one more study, mean value of 10.50 mg/L for the summer period was observed which is not align with these finding and for the rainy season 70 mg/L was observed ^76^. Also, Magaji and Adakayi^79^ reported variation among different season which have a mean value of 79.8 mg/L and 45 mg/L for the summer and rainfall period. A study by Isaac et al.,^87^ observed a comparatively higher value of sulphate in water bodies ranging from 111.03 to 198.76 mg/L. The source of Sulphate in water bodies is due to the mineral rocks and agriculture effluents.

The lack of significant seasonal shifts in sulphate and chloride levels suggests that their inputs are likely chronic and geogenic, or stem from sustained agrochemical usage rather than episodic pollution events. While concentrations remain within safe irrigation limits, their continuous presence could lead to cumulative salinity stress on crops and progressive soil quality decline, particularly in regions with low natural drainage or high evapotranspiration. The findings support the implementation of integrated soil–water management plans, including the promotion of salt-tolerant crops in susceptible zones and periodic soil salinity testing to anticipate and mitigate any long-term impacts on agricultural sustainability.

*Heavy metals* Heavy metal contamination of irrigation water is a major environmental threat because firstly its nature is non-biodegradable and secondly, it’s potential accumulation in numerous body parts. It has been observed that irrigation wastewater causes the deposition of heavy metal in agricultural soils and vegetables consumed by the residents get exposed to these excessive heavy metals^92^. In the current study, Aluminium (Al), values observed were high for the pre-rainfall period. Also, for the post-rainfall period, Al values of some sites were not within the recommended concentration. The mean concentration for dry season for Aluminium (Al) was 0.659 mg/L and the post rainfall season concentration was 2.207 mg/L. For the Iron (Fe), similar observation was recorded as the values of iron were very high during the rainfall period. The mean value of iron (Fe) for rainfall period was 2.090 mg/L and for the summer period concentration reported was 0.982 mg/L. Molekoa et al.^93^ observed a comparatively lower concentration of Iron where the lowest values recorded was 0.0069 mg/L and the highest value observed in the study was1.87 mg/L which matches with the current result of the study. And for the copper (Cu), the observed mean value for rainy season was 0.124 mg/L and for the summer period, the concentration was 0.042 mg/L. In one study by Li et al.^94^ the values of copper ranged from 0.0008 to 0.00487 mg/L which is lower than the observed value in this study. Similarly, Pb, Cr, Zn values observed for the rainfall period were very high for few sites. The mean values of Pb, Cr and Zn concentration for rainfall period were 0.063 mg/L, 0.054 mg/L and 0.45 mg/L and for the dry season, the concentration were 0.062 mg/L, 0.017 mg/L and 0.50 mg/L. Compared with previous study on water body, the values of zinc ranging higher than that reported in a study by Xie and Ren^95^ where the observed mean value was 0.01 mg/L. In the present study, no significant spatial variation for Heavy metals observed. However, a major fluctuation of values was observed for different season. Similar study was conducted in the past where it was observed that the heavy metals values were within the acceptable FAO limits but the seasonal variation for various parameter was notably significant (*p* value ˂ 0.05)^55^. Table 8 present the mean values of heavy metals parameters for all the sites.

**Table 8:** Descriptive statistics of metals at the studied sites.

Sites		Heavy Metals mg ${L}^{-1}$					
		${Al}^{++}$	${Cr}^{++}$	${Cu}^{++}$	${Fe}^{++}$	${Pb}^{+}$	${Zn}^{++}$
Site 1	Mean	1.395	0.027	0.093	1.097	0.044	0.355
Site 2	Mean	1.230	0.021	0.076	1.193	0.035	0.490
Site 3	Mean	1.492	0.018	0.158	1.224	0.039	0.418
Site 4	Mean	1.318	0.022	0.098	1.355	0.060	0.419
Site 5	Mean	1.501	0.023	0.083	1.428	0.041	0.353
Site 6	Mean	1.309	0.035	0.056	1.387	0.039	0.426
Site 7	Mean	1.250	0.042	0.061	1.281	0.044	0.340
Site 8	Mean	1.421	0.039	0.074	1.239	0.036	0.228
Site 9	Mean	1.154	0.016	0.093	1.080	0.066	0.306
Site 10	Mean	1.207	0.016	0.057	1.060	0.104	0.352
Site 11	Mean	0.715	0.014	0.017	0.773	0.024	0.168

The prevalence of heavy metals such as cadmium (Cd), chromium (Cr), and manganese (Mn) can be attributed to a combination of geogenic and anthropogenic factors. Geologically, the canal and surrounding areas lie in a terrain where mineral-rich lithologies, especially those containing iron-manganese oxides and ultramafic rocks, may naturally release trace metals through weathering processes. However, anthropogenic inputs appear more dominant. Agricultural runoff, especially from areas with intensive use of phosphate fertilizers and pesticides, has been widely reported as a major source of Cd and Cr contamination in canal systems. Similarly, the use of manganese-based fungicides and leaching from nearby industrial activities could contribute to elevated Mn levels. The hydrological regime of the canal—characterized by intermittent flow and stagnation—may also exacerbate the mobilization and accumulation of these metals by influencing redox conditions and sediment interactions. These interpretations align with findings from similar agro-industrial watersheds^104^, where mixed land use and poor waste management practices have led to heavy metal enrichment in water bodies.

The observed seasonal fluctuations in metals such as Al, Fe, and Cu closely align with agricultural activity patterns in the region. During the pre-monsoon and early monsoon periods, increased use of fertilizers and agrochemicals, combined with surface runoff from agricultural fields, likely contributes to elevated metal concentrations in canal water. Post-monsoon dilution tends to reduce these levels temporarily. Furthermore, sediment mobilization during peak flows can resuspend previously deposited metal-laden particulates, exacerbating seasonal peaks. Mitigation is feasible through land-use and irrigation management strategies such as constructing vegetative buffer zones, promoting controlled fertilizer application, adopting drip irrigation to reduce runoff, and ensuring proper drainage infrastructure. Additionally, encouraging soil testing and precision agriculture could limit excess agrochemical usage, directly reducing metal inputs into the water system. These practices would not only help stabilize seasonal variability but also promote sustainable farming and water quality conservation in the long term.

**Fig. 6:**
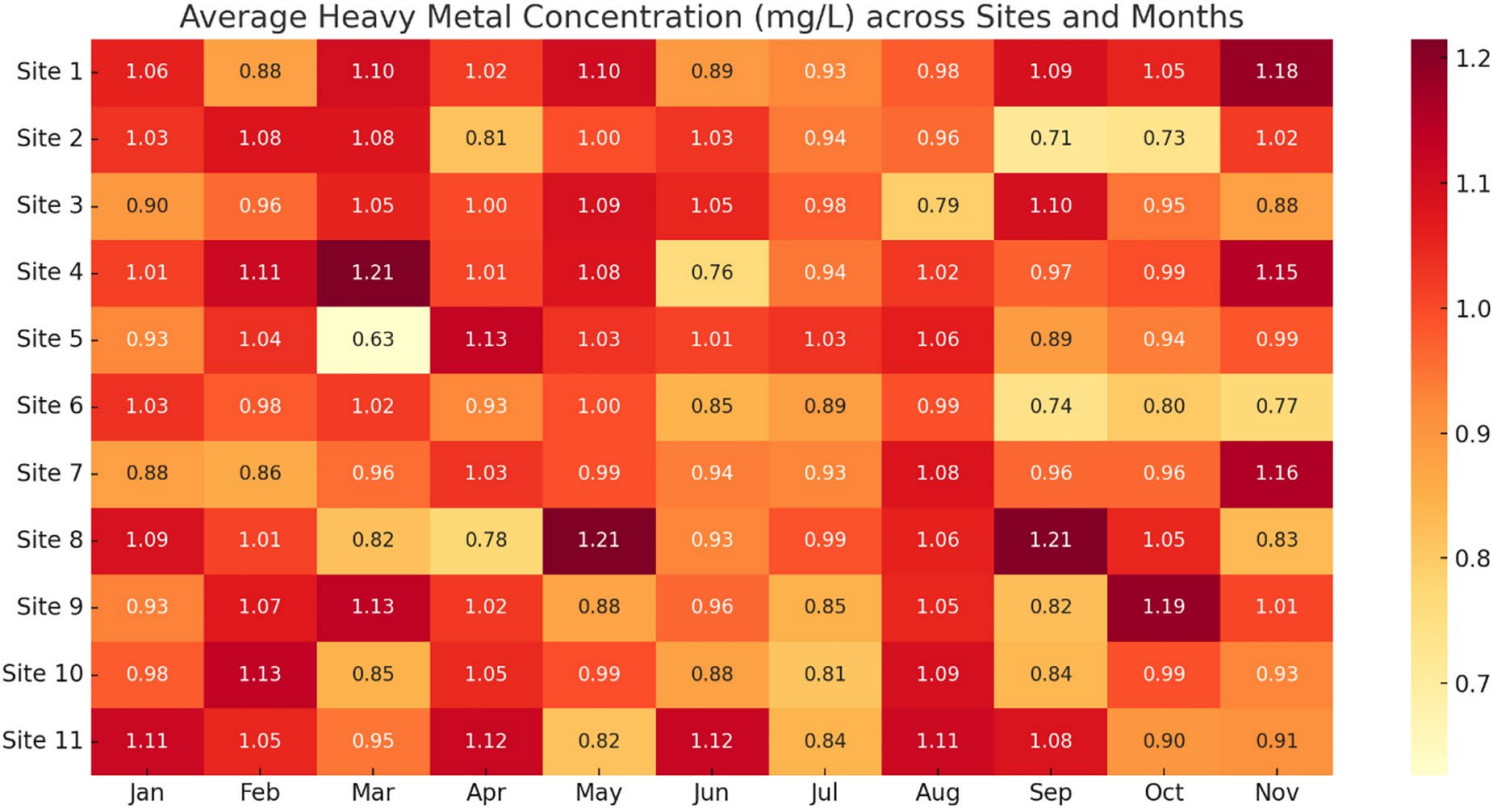
Average heavy metal concentration across sites and methods.

Figure 6 captures the spatial and temporal distribution of heavy metals (Al, Fe, Zn, Cu, Cr, Pb) averaged monthly across all sites. Peaks in metal concentration are noticeable during the early monsoon months (June–August), likely due to increased surface runoff and sediment mobilization. Sites 1, 3, and 5 show higher metal loads across multiple months, indicating consistent exposure or proximity to pollution sources like agricultural runoff or domestic waste inputs.

### Outcomes of principal component analysis

**Fig. 7:**
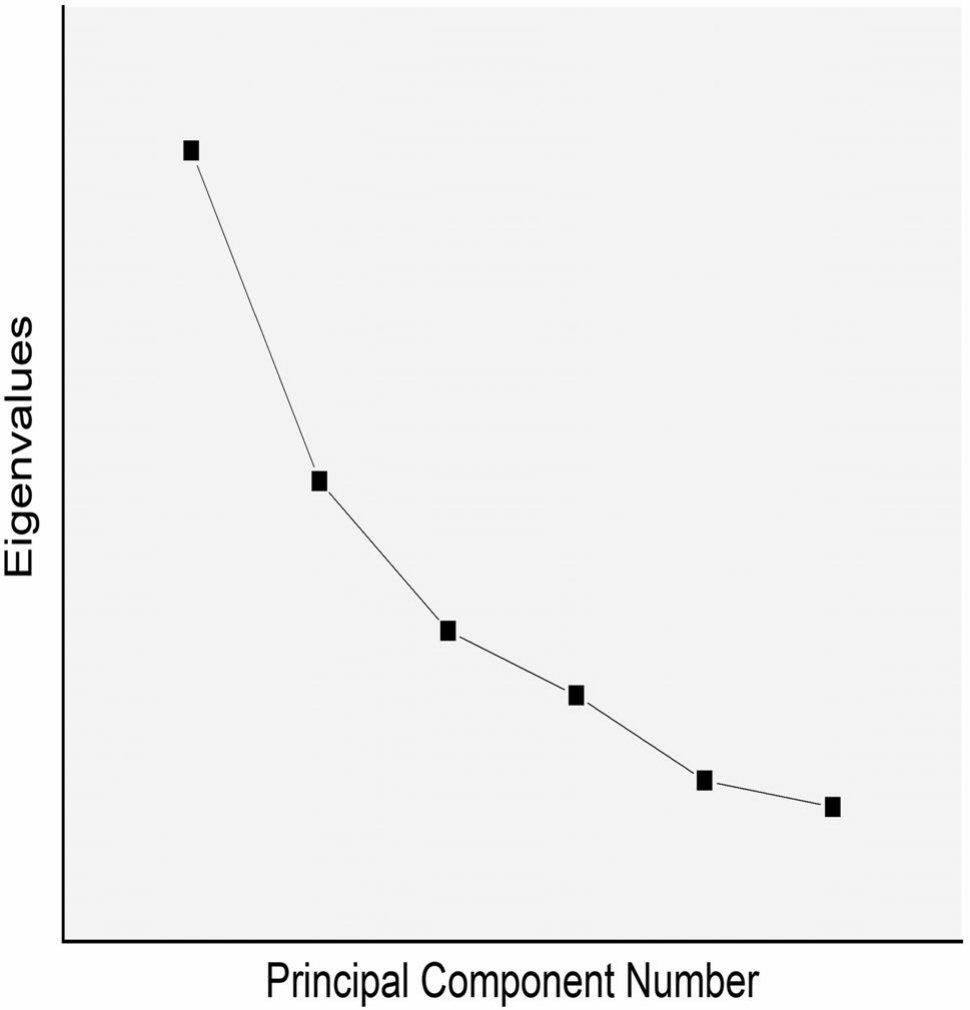
Scree Plot depicting eigenvalues.

**Fig. 8:**
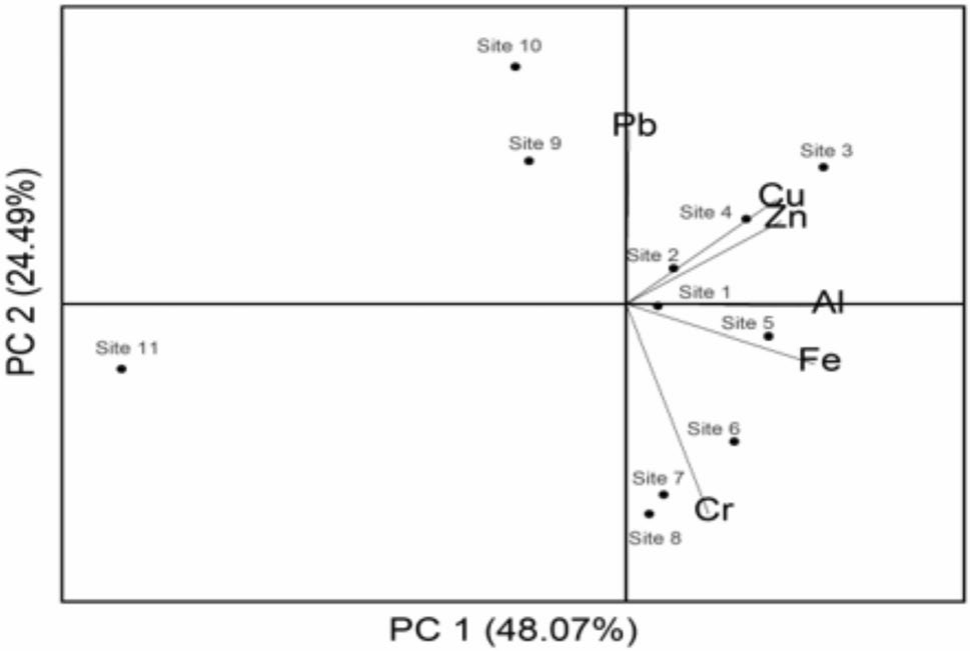
Biplot of the water quality variables (Heavy metals).

The sources of heavy metals in the canal water body were investigated by applying PCA with varimax rotation. The first PC1 can explain above 48.07% of the total variance and it has strong loading on Al (r = 0.502), Fe (r = 0.511). Whereas the second PC can explain 24.49% of the total variance and it has strong loading on Cu (r = 0.664). Consequently, this factor captures the non-point discharges, most of which are from the geological and anthropogenic activities. Table 9 presents the details of the loadings or the coefficients that are multiplied by the variables to obtain the PCs. The number of components can be determined using the scree plot are shown in Fig. 7 and the biplot of the water quality variables is shown in Fig. 8. There is a major break observed in the scree plot after the second component. The elevated levels of heavy metals in the water bodies may be due to agricultural lands and small industrial effluents^96^. There are other authors who have noted that anthropogenic contribution in water bodies, particularly from agricultural activities such as the application of pesticides and fertilizers in water bodies^10–12,97^. In one study it is clearly evident that the value of the various parameters in water bodies is due to geological process, indicating geogenic sources^62^.

**Table 9:** Coefficients of heavy metal variables for principal components.

Heavy Metal parameters	Principal components	
	PC1	PC2
Al	0.5025	0.2640
Cr	0.3733	− 0.5762
Cu	0.1445	0.6645
Fe	0.5111	− 0.0801
Pb	− 0.5698	− 0.0239
Zn	0.0354	0.3867

The PCA results demonstrate the value of dimensionality reduction in revealing dominant pollution patterns and parameter interlinkages. The grouping of Al, Fe, and Cu under major components suggests shared sources, possibly related to agro-industrial discharges or sediment mobilization. These associations can aid in hypothesizing pollutant transport mechanisms and in designing simplified, cost-effective monitoring programs focused on a few key indicators. By capturing most of the variance with two principal components, this analysis validates the use of PCA in designing early-warning systems or rapid water quality assessment protocols, which are crucial for real-time canal management and regulatory interventions.

### Outcomes of clustering analysis

**Fig. 9:**
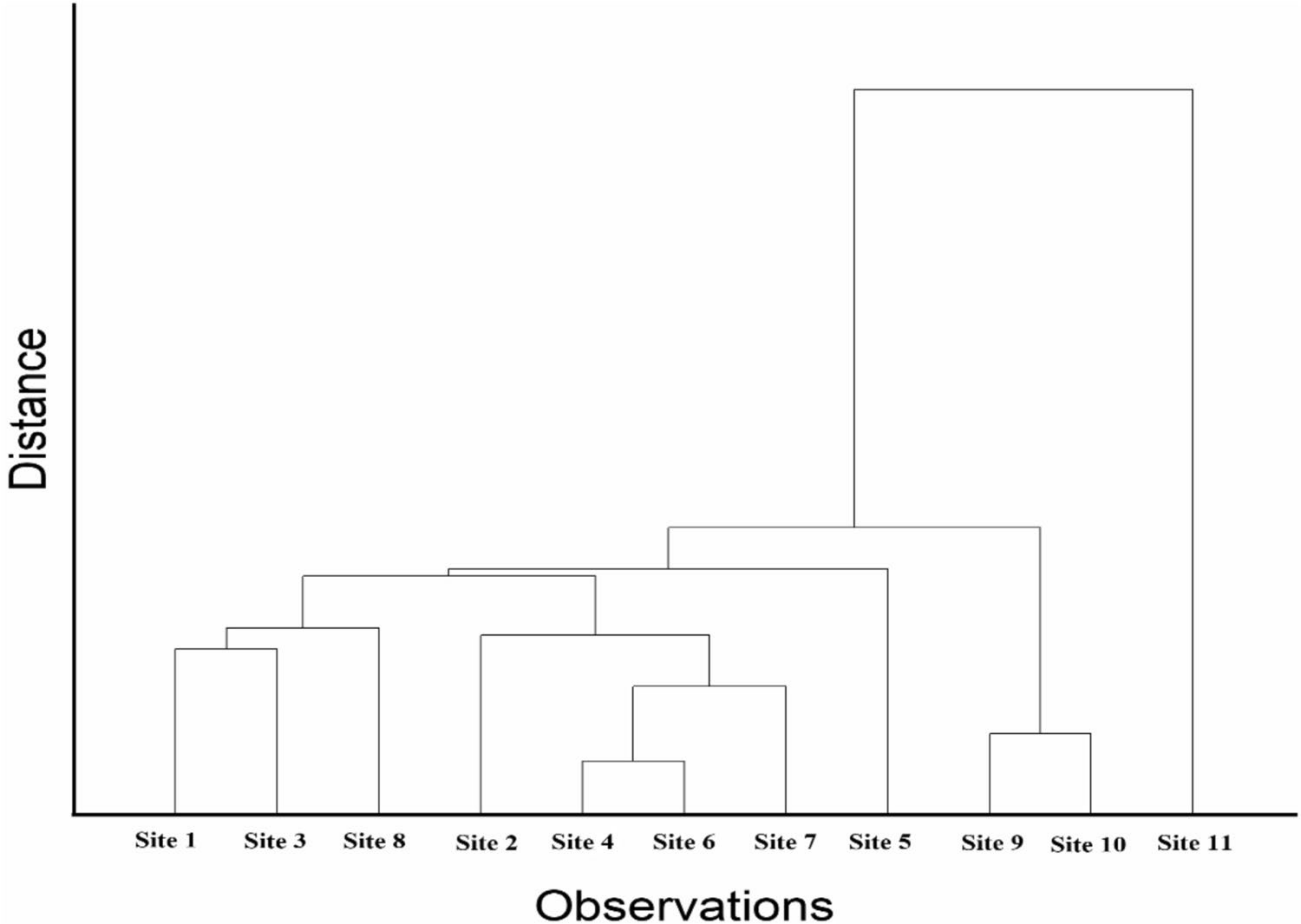
Q-HCA showing clusters of sampling sites.

The classification of datasets into clusters depends on the homogeneity/non-homogeneity among the datasets^98^. It can be observed through Q-HCA (Fig. 9) that the sites are divided into two major clusters with Cluster 1 including site 11. The areas of this cluster are generally well endowed with Aluminium and Iron. The sites however are placed in the rural which is prone to anthropogenic stresses due mainly to agricultural and human activities. The high value of heavy metals in this cluster is believed to be detrimental to the health of crops and humans, as bathing activities in the canal water was observed during the study. Cluster two has two sub-cluster which includes site 1, site 3, site 8, in one sub-cluster. These sites are particularly characterized with the highest value of Al (1.491 mg L^−1^), Fe (1.239 mg L^−1^) and Zn (0.417 mg L^−1^). The second sub-cluster includes site 2, site 4, site 6 and site 7 (Fig. 7). These sites are particularly characterized with the highest value of Al (1.318 mg L^−1^), Fe (1.386 mg L^−1^) and Zn (0.489 mg L^−1^). The site 7 of canal system is close to the pump house. The water is scheduled in 15 days duration in this study area and for the rest of the time-period there’s no water in canal. At site 7 and site 6garbages of plastic waste and other waste materials were observed as this site is close to the human population. Also, at site 8, actions like bathing, washing of clothes was observed during the study. As a result, a lot of pollution is caused in the canal water.

**Fig. 10:**
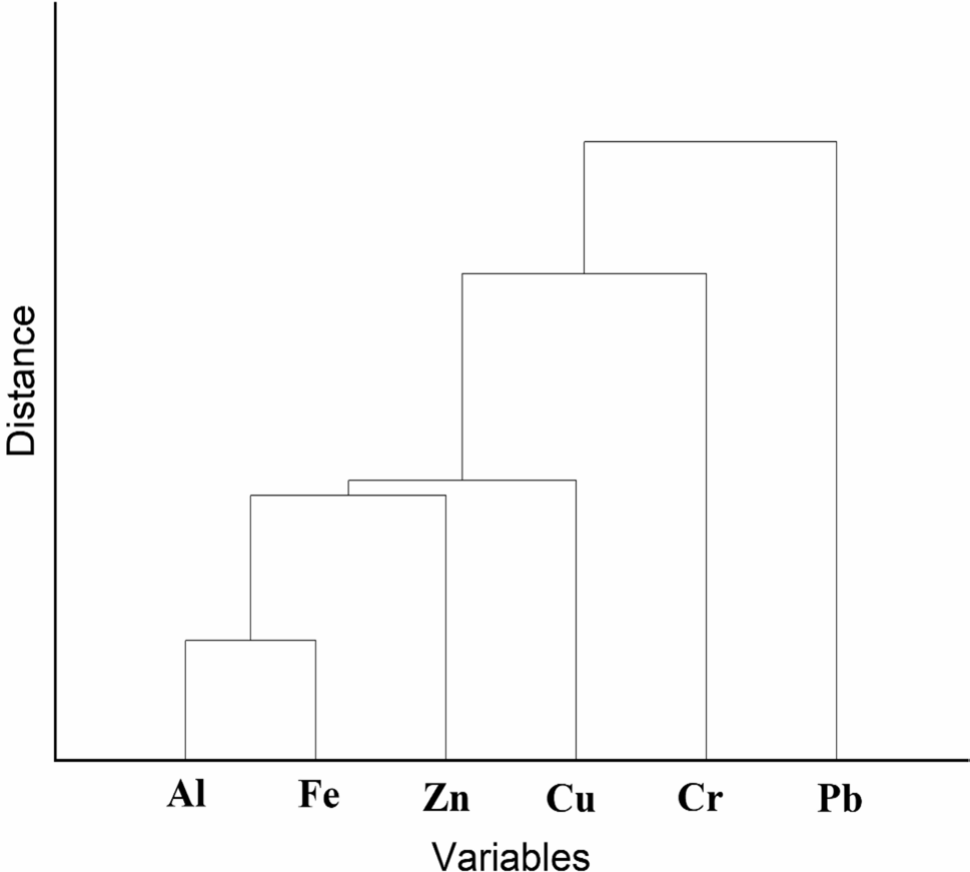
R-HCA showing clusters of analyzed heavy metals.

Two major clusters of heavy metals are evident in the R-HCA (Fig. 10). First consist of Pb and cluster 2 has two sub-cluster which consist of Al, Fe and Zn in one sub-cluster and Cr and Cu in other sub-clusters. The Aluminium and Iron parameters have a close linkage may be due to heavy rainfall runoff laden with agriculture effluents such as pesticides and other small industries pollutants. Figure 9 clearly depicts the challenges of the study area using images.

The cluster analysis outcomes clearly distinguish between zones of varying pollution pressure within the canal network, enabling a spatially informed risk categorization. The grouping of sites with higher metal concentrations suggests localized pollution hotspots, likely influenced by nearby settlements, agricultural runoff, or direct waste disposal. Similarly, the clustering of metal parameters indicates shared pathways and behaviors in the aquatic environment. These insights provide a practical framework for implementing spatially targeted monitoring and remediation programs, allowing water managers to prioritize intervention in high-risk stretches and better allocate resources for pollution control and ecological restoration.

The spatial grouping of sampling sites into distinct clusters based on heavy metal concentrations closely aligns with studies such as Srinivas et al.^22^, which also employed hierarchical clustering to identify contamination hotspots in canal and river systems. In this study, Cluster 1 was defined by elevated Al and Fe levels and found to be strongly influenced by nearby anthropogenic activities such as bathing, solid waste disposal, and agricultural runoff. Similar spatial patterns were observed in studies across developing regions where rural settlements interface directly with water bodies (Wang et al., ^55^; Khalid et al., ^99^). The clustering of Al, Fe, and Zn in sub-clusters is consistent with findings from Xie and Ren^95^, who attributed such associations heavy metals in rivers in the antimony capita of Xikuangshan. Our results not only reinforce the utility of cluster analysis in segregating pollution risk zones but also underscore the need for micro-level interventions, as high-pollution clusters were found adjacent to specific human activities—an insight also highlighted by Mechal et al.^83^.

The identification of distinct pollution clusters enables spatially focused decision-making, allowing regulatory bodies to prioritize high-risk zones rather than applying uniform interventions across the entire canal system. For instance, clusters showing elevated levels of Al, Fe, and Zn—particularly near sites with known anthropogenic pressures such as waste disposal and agricultural runoff—can be targeted for localized remediation measures like buffer strips, regulated discharge controls, or sediment removal. These hotspot-specific insights also support the design of cost-effective monitoring frameworks, where fewer but strategically placed sensors can capture key pollution trends. At the policy level, such clustering outcomes can inform zoning regulations, targeted awareness campaigns, and integration of pollution mitigation plans within broader watershed management policies. Ultimately, the ability to spatially isolate pollution drivers enhances the precision and impact of both regulatory and community-driven interventions.

The application of Principal Component Analysis (PCA) in this study allowed for the extraction of dominant pollution patterns by identifying intercorrelated heavy metals contributing most to overall variance. For instance, the grouping of Al and Fe in PC1 suggested sediment-associated inputs likely influenced by agro-industrial runoff, while Cu loading in PC2 pointed toward localized anthropogenic activity. This dimensionality reduction isolated the most influential variables, aiding source attribution. Hierarchical Cluster Analysis (HCA) further complemented this by organizing both sampling sites and metal parameters into clusters based on similarity metrics. The Q-mode HCA revealed spatially coherent clusters of sites with elevated contamination, while R-mode HCA grouped metals with shared transport or origin characteristics. This dual application enabled the clear differentiation of pollution sources and spatial variability, making it a robust approach for targeted monitoring and management strategies.

### Irrigation water quality index (IWQI) assessment

In the current study, The FA was applied to 6 variables, including TDS, EC, SAR, %Na, MH, KI. The KMO adequacy test and the Bartlett sphericity test indicate that the value is greater than 0.5 and lower than 0.001, respectively. Hence, the performed factorial model is adequate for this study. Based on the eigenvalue, which should be greater than 1, factors are selected (Table 10). The first factor, C1, expressed more than 63.96% of the total variance. The second factor, C2, has a variance of 19.503%. However, C3 and C4 factors have a variance of 12.420% and 3.868%, respectively.

**Table 10:** Percentage variance of selected principal components.

Component	Eigenvalue	% of variance	% of cumulative variance
1	3.838	63.966	63.966
2	1.170	19.503	83.469
3	0.745	12.420	95.889
4	0.232	3.868	99.757
5	0.011	0.188	99.945
6	0.003	0.055	100.00

In the current research, the computation of the IWQI is based on the parameters that have a good load in the first component such as TDS, EC, MH, KI, %Na, SAR. C1 is considered as the most significant component that explains the global variability in water quality. Furthermore, the weight values are estimated based on the variance of the first component related to its explainability towards each parameter. The normalized weight values are presented in Table 11.

**Table 11:** Calculated relative weight of each parameter.

Parameter	SAR	Na%	MH	KI	EC	TDS
Weight	0.190	0.216	0.142	0.220	0.093	0.139

The IWQI computed lowest value observed was 67.27, and samples fell into the ‘Good’ and ‘Very Good’ categories, indicating that water quality is generally suitable for irrigation purposes. However, the presence of heavy metal concentrations in the water body during the rainfall months and post-rainfall period should be a focus of concern, as it may pose risks to soil health, crop productivity.

The IWQI classification indicates that while canal water is generally suitable for irrigation, there exist sub-threshold conditions — particularly related to sodium and heavy metals — that could compromise long-term soil fertility and crop productivity if unaddressed. The use of factor analysis in weighting enhances the robustness of IWQI results and minimizes the subjectivity inherent in traditional index models. However, the seasonal sensitivity of IWQI outcomes suggests the need for dynamic thresholding and adaptive irrigation planning. The findings advocate for incorporating such indices into regular agricultural extension programs to support farmers in making water-use decisions that align with both crop requirements and soil conservation goals (Table 12).

**Table 12:** Matrix of correlation coefficients for various parameters.

	pH	Ec	Do	TDS	${\text{Ca}}^{++}$	${\text{Mg}}^{++}$	${\text{Na}}^{+}$	${\text{K}}^{+}$	${\text{Cl}}^{-}$	${{\text{So}}_{4}}^{2-}$
pH	1.00									
Ec	− 0.50	1.00								
Do	0.13	− 0.01	1.00							
TDS	0.22	0.42	− 0.27	1.00						
${\text{Ca}}^{++}$	− 0.35	0.75	0.22	0.03	1.00					
${\text{Mg}}^{++}$	− 0.04	− 0.41	0.55	− 0.87	0.04	1.00				
${\text{Na}}^{+}$	− 0.39	0.00	− 0.07	− 0.20	0.26	0.13	1.00			
${\text{K}}^{+}$	− 0.24	0.65	0.19	0.50	0.62	− 0.24	0.39	1.00		
${\text{Cl}}^{-}$	0.17	− 0.31	0.70	− 0.64	0.02	0.72	− 0.28	− 0.42	1.00	
${{\text{So}}_{4}}^{2-}$	0.08	− 0.37	0.19	− 0.54	− 0.34	0.38	− 0.25	− 0.78	0.62	1.00

The IWQI assessment in this study revealed that several sites within the canal network fall under “high restriction” to “unsuitable” categories for irrigation use, primarily due to elevated concentrations of Al, Fe, and Zn. These findings are consistent with those reported by Luo et al.^100^ in contaminated irrigation canals of South Asia, where metal-laden runoff severely compromised irrigation suitability. Moreover, the spatial variability observed here corresponds with previous research indicating strong links between land use, surface runoff pathways, and IWQI values (Talpur et al.,^101^; Baloch et al.,^102^). Unlike some earlier works that focused solely on SAR or EC, our study integrates heavy metals into the IWQI framework, offering a more holistic risk assessment for agriculture. This approach builds upon methodologies outlined by Gad et al.^103^, who emphasized the growing relevance of multi-parameter IWQI evaluations in regions facing compound anthropogenic pressures. Thus, our analysis adds to the body of evidence supporting the incorporation of trace metals in irrigation suitability assessments under changing environmental conditions.

**Fig. 11:**
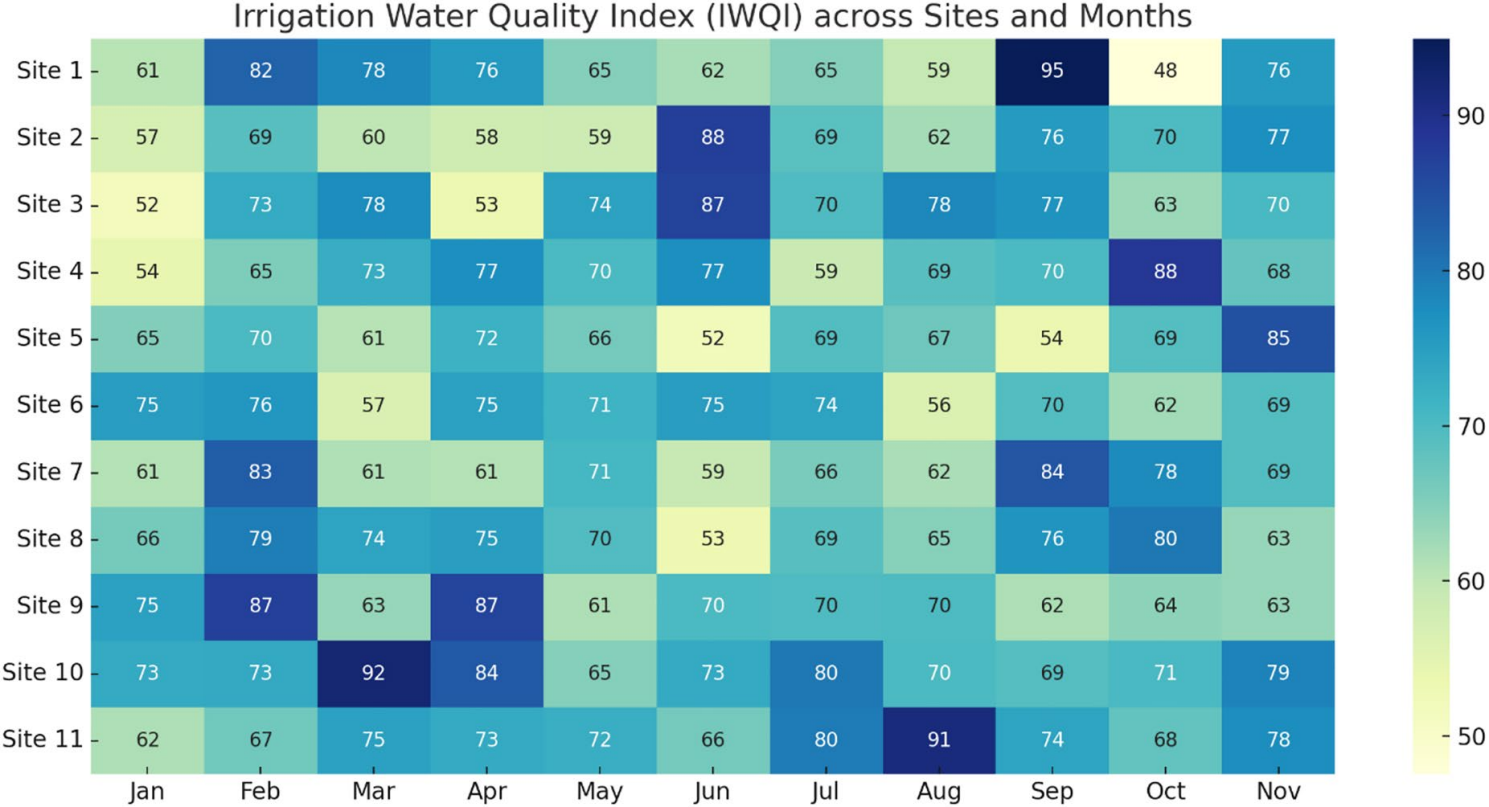
IWQI across sites and months.

The IWQI scores (Fig. 11) highlight water suitability for irrigation over the 11-month period. Sites like 2 and 3 exhibit better water quality in post-monsoon months, while scores drop in June–July, possibly due to contamination peaks. Conversely, Site 1 maintains moderate IWQI levels but sees a spike in September, suggesting episodic improvement in water conditions—perhaps due to system flushing or temporary reduction in pollutant inflows.

### Farmer survey outcomes

Around 479 people were sampled via the offline survey. For the pre-rainfall period, the farmers rated the canal water quality appearance to be in the range of '2'–‘5’ on a 5-part Likert scale. For the post-rainfall period, the farmers rated the canal water quality appearance to be in the range of ‘2’–‘5’ on a 5-part Likert scale. For the aesthetic quality of canal water, the pooled sample average appearance receives less poor rating of canal water for the rainfall period and post-rainfall. Similarly, for smell ratings, farmers gave slightly lower ratings for the smell of canal water for the post-rainfall period compared to pre-rainfall period.

**Fig. 12:**
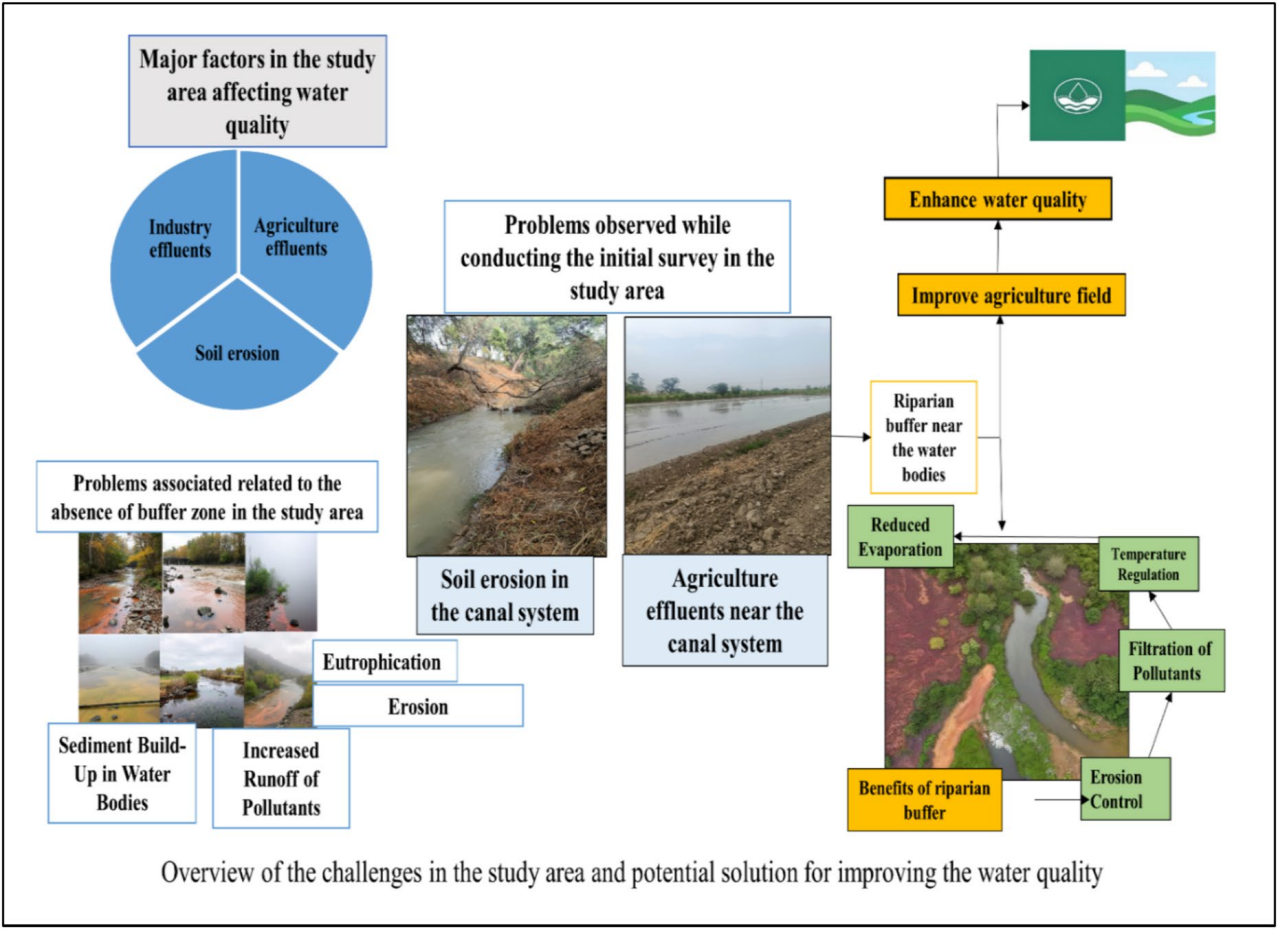
Challenges in the study area and possible solution for improving the canal water quality.

When asked about the awareness they have about the impact of water quality on crops, many (78.91%) agreed that poor water quality would affect the crops. Also, During the survey it was asked whether they observed the decline in water quality or not. Majority of the farmers (89.35%) agreed that there is a decline in water quality in terms of appearance and smell. When asked about whether the farmers have information related to improving the water quality for their agriculture fields, a large percentage (53.02%) agreed that they have less knowledge related to the preservation of water quality. Following the survey, we asked whether they have any information about riparian buffer, (43.84%) farmers do have information about riparian buffer and (32.56%) farmers agreed that it can improve the surface water quality. Figure 12 represents the challenges in the study area and possible solution for improving the canal water quality.

Table 13 correlates the farmers’ qualitative perception about the water quality and proposed solutions. The perceptions of the farmers very much corroborate the observed water quality and thus necessitate prompt action from the local irrigation department.

**Table 13:** Correlating farmer’s perceptions with the water quality data of the study area.

Sampling Station	Water Quality Parameters	Farmer Perception	Proposed Solution based on the analysis
Station 1	pH: 7.2, EC: 750 µS/cm, TDS: 460 mg/L, TH: 250 mg/L, Ca: 58 mg/L, Mg: 25 mg/L, Na: 140 mg/L, K: 5 mg/L, Cl: 80 mg/L, SO₄: 130 mg/L	No major issues, occasional salty taste	Periodic water testing, maintain EC below 800 µS/cm
Station 2	pH: 6.9, EC: 820 µS/cm, TDS: 510 mg/L, TH: 270 mg/L, Ca: 62 mg/L, Mg: 28 mg/L, Na: 155 mg/L, K: 6 mg/L, Cl: 85 mg/L, SO₄: 140 mg/L	Leaves turning pale, slight skin irritation	Use of gypsum, awareness on water pH management
Station 3	pH: 6.8, EC: 890 µS/cm, TDS: 560 mg/L, TH: 290 mg/L, Ca: 65 mg/L, Mg: 30 mg/L, Na: 170 mg/L, K: 7 mg/L, Cl: 90 mg/L, SO₄: 150 mg/L	Wilting in vegetables, bitter taste reported	Treated water for irrigation, avoid sensitive crops
Station 4	pH: 7.0, EC: 710 µS/cm, TDS: 440 mg/L, TH: 240 mg/L, Ca: 55 mg/L, Mg: 24 mg/L, Na: 130 mg/L, K: 5 mg/L, Cl: 75 mg/L, SO₄: 120 mg/L	Water suitable for crops, no complaints	Continue monitoring, promote water-efficient crops
Station 5	pH: 6.7, EC: 950 µS/cm, TDS: 600 mg/L, TH: 310 mg/L, Ca: 68 mg/L, Mg: 32 mg/L, Na: 180 mg/L, K: 7 mg/L, Cl: 95 mg/L, SO₄: 160 mg/L	Poor germination, itching after use	Improve water blending, use mild filtration
Station 6	pH: 7.1, EC: 770 µS/cm, TDS: 470 mg/L, TH: 260 mg/L, Ca: 60 mg/L, Mg: 26 mg/L, Na: 145 mg/L, K: 5 mg/L, Cl: 82 mg/L, SO₄: 135 mg/L	Yield drop in groundnut, rough texture on skin	Furrow irrigation, promote calcium and magnesium balance
Station 7	pH: 7.3, EC: 690 µS/cm, TDS: 430 mg/L, TH: 230 mg/L, Ca: 52 mg/L, Mg: 22 mg/L, Na: 125 mg/L, K: 4 mg/L, Cl: 70 mg/L, SO₄: 110 mg/L	Germination delay, but usable water	Delay planting till initial rainfall, crop rotation
Station 8	pH: 6.9, EC: 880 µS/cm, TDS: 550 mg/L, TH: 285 mg/L, Ca: 64 mg/L, Mg: 29 mg/L, Na: 165 mg/L, K: 6 mg/L, Cl: 88 mg/L, SO₄: 145 mg/L	Salt stains on utensils, crop yellowing	Awareness programs, introduce filtration units
Station 9	pH: 6.6, EC: 920 µS/cm, TDS: 580 mg/L, TH: 300 mg/L, Ca: 67 mg/L, Mg: 31 mg/L, Na: 175 mg/L, K: 7 mg/L, Cl: 93 mg/L, SO₄: 155 mg/L	Slight dryness in crops, bitter water	Blend with cleaner source, improve soil health
Station 10	pH: 7.0, EC: 740 µS/cm, TDS: 450 mg/L, TH: 245 mg/L, Ca: 57 mg/L, Mg: 25 mg/L, Na: 135 mg/L, K: 5 mg/L, Cl: 78 mg/L, SO₄: 125 mg/L	Acceptable quality, minor chlorosis	Promote hardy crops, seasonal monitoring
Station 11	pH: 7.2, EC: 700 µS/cm, TDS: 420 mg/L, TH: 225 mg/L, Ca: 50 mg/L, Mg: 20 mg/L, Na: 120 mg/L, K: 4 mg/L, Cl: 68 mg/L, SO₄: 105 mg/L	No visible impact, water is clear	Maintain practices, periodic chemical assessment

### Suggesting water treatment solutions for the study area

The study’s findings offer valuable insights for improving irrigation water management in semi-arid regions. The spatial identification of contamination hotspots and seasonal trends in metal concentrations can help water managers prioritize site-specific interventions rather than adopting uniform treatment strategies. For instance, at locations where heavy metal loads consistently exceed safe thresholds, constructed wetlands or phytoremediation using metal-tolerant plant species could be implemented as cost-effective treatment options.

Furthermore, recognizing the role of agricultural runoff in contaminant loading, the study underscores the importance of controlled fertilizer and pesticide application, use of biofertilizers, and promotion of organic farming in vulnerable stretches of the canal. Rain-triggered peaks in pollution also point to the need for buffer strips, sediment traps, and riparian vegetation to intercept contaminants before they reach water bodies.

From a governance perspective, the research supports the development of early warning systems and seasonal water quality monitoring programs using simple indicators (e.g., IWQI), which can help farmers make informed irrigation decisions. These sustainable practices, informed by empirical evidence, would help protect crop health, reduce soil degradation, and conserve long-term water quality in semi-arid agro-ecosystems.

## Conclusions

According to the classification of irrigation water quality index (IWQI), the regional variance of physico-chemical parameters between the various research sites was statistically insignificant. Also, seasonal variation of physico-chemical parameters was statistically significant. For heavy metal like Iron (Fe), copper (Cu), and aluminium (Al) similar observation was recorded as the values of were very high for few sites during the rainfall and post rainfall period. Also, dissolved oxygen (DO) values were very low for summer period (Mean value of DO = 4.40 mg/L). Also, potassium (${\text{K}}^{+}$) values measured in the lab were at elevated level and were not within the FAO permissible limit (2 mg/L). Therefore, this study recommended conducting more studies in this area to shade more light on the area on a regular and continuous basis in future works to monitor changes in water quality and avoid canal water pollution. Also, the survey developed in this paper represents the farmer’s perception of canal water quality. It gives farmer’s perception of water quality for the agriculture purposes. Farmers gave poor rating during post-rainfall period. The data collected of farmers perception of canal water quality is matching with the collected physico-chemical data as it is observed that the values of heavy metals showed a significant seasonal variation between different seasons (pre rainfall and post rainfall period). Also, while having an open discussion with farmers it has been found out that farmer’s lack of knowledge about preservation techniques for water quality.This study introduces an advance method for identifying key sources in canal water body using multivariate cluster analysis. It utilizes advanced visual tools which include dendrograms, scree plots and biplots. These visualizations effectively highlights the relationships between different pollutants and their potential sources, enabling the classification of pollutants. By simplifying complex environmental data, this approach offers valuable insights to pollution control agencies and policymakers, enabling effective interventions to address pollution sources.

## Data Availability

The datasets analyzed during the current study are not publicly available due to ethical concerns regarding privacy and the protection of personal data but are available from the corresponding author on reasonable request. All methods including questionnaire survey were carried out in accordance with relevant guidelines and regulations by the Department of Civil Engineering, Birla Institute of Technology and Science, Pilani, India. All experimental protocols (questionnaire survey) were approved by the Department of Civil Engineering, Birla Institute of Technology and Science, Pilani, India.
